# A heavy metal P-type ATPase OsHMA4 prevents copper accumulation in rice grain

**DOI:** 10.1038/ncomms12138

**Published:** 2016-07-08

**Authors:** Xin-Yuan Huang, Fenglin Deng, Naoki Yamaji, Shannon R.M. Pinson, Miho Fujii-Kashino, John Danku, Alex Douglas, Mary Lou Guerinot, David E. Salt, Jian Feng Ma

**Affiliations:** 1 Institute of Biological and Environmental Sciences, University of Aberdeen, Aberdeen AB24 3UU, UK; 2 Institute of Plant Science and Resources, Okayama University, Kurashiki 710–0046, Japan; 3 USDA–ARS Dale Bumpers National Rice Research Center, Stuttgart, Arkansas 72160, USA; 4 Department of Biological Sciences, Dartmouth College, Hanover, New Hampshire 03755, USA

## Abstract

Rice is a major source of calories and mineral nutrients for over half the world's human population. However, little is known in rice about the genetic basis of variation in accumulation of copper (Cu), an essential but potentially toxic nutrient. Here we identify *OsHMA4* as the likely causal gene of a quantitative trait locus controlling Cu accumulation in rice grain. We provide evidence that OsHMA4 functions to sequester Cu into root vacuoles, limiting Cu accumulation in the grain. The difference in grain Cu accumulation is most likely attributed to a single amino acid substitution that leads to different OsHMA4 transport activity. The allele associated with low grain Cu was found in 67 of the 1,367 rice accessions investigated. Identification of natural allelic variation in *OsHMA4* may facilitate the development of rice varieties with grain Cu concentrations tuned to both the concentration of Cu in the soil and dietary needs.

## Abstract (web summary)

Copper (Cu) is an essential mineral nutrient but high concentrations in rice grain can cause toxicity. Here the authors provide evidence that natural variation in rice grain Cu concentration is caused by altered sequestration of Cu into root vacuoles due to a single amino acid substitution in the OsHMA4 transporter.

## Graphical abstract

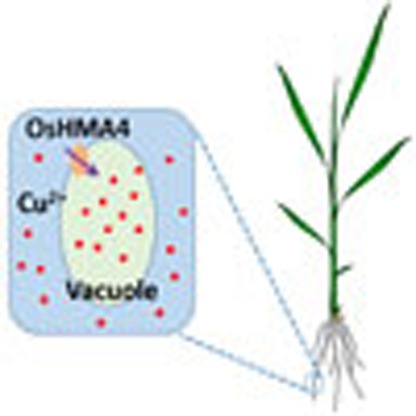

Copper (Cu) is an essential micronutrient for all living organisms. In plants, Cu acts as a redox-active cofactor and participates in multiple biological processes such as photosynthesis, respiration, cell wall remodelling, oxidative stress resistance and ethylene perception^1^,^2^. Cu deficiency reduces growth rates, seed set and yield due to impaired photosynthesis and pollen fertility. Cu also plays important roles in human health as an enzymatic cofactor involved in cellular respiration, free radical detoxification, pigmentation, neuron development, connective tissue formation and iron transport^3^,^4^. Currently, more than two billion people worldwide suffer from micronutrient deficiencies such as iron (Fe), zinc (Zn) and Cu^5^,^6^. Cu deficiency causes immune defects and anaemia^3^,^7^. The estimated average requirement for Cu is 260–685 μg per day for children depending on age, 700 μg per day for adults and 1,000 μg per day for women during pregnancy and lactation^8^. In the human diet, Cu is enriched in meat, fish and nuts. However, such food is not usually available to most populations suffering from micronutrients deficiencies. Therefore, biofortification to increase Cu in staple foods such as rice is one approach to provide the minimum amount of dietary Cu for these populations.

On the other hand, Cu is toxic when present in excess, mainly due to its role in generating highly reactive oxygen species that cause cellular damage^1^,^2^,^3^. Due to the over use of Cu-containing fungicides, and the release of Cu in industrial wastewater and from mining activities, Cu contamination of cultivated soils and irrigation waters has become problematic in certain regions. For example, Cu is ranked as the fourth most contaminating heavy metal of agricultural lands in China^9^. Thus, it is important to develop rice (*Oryza sativa* L.) cultivars that are both tolerant to Cu and that can exclude excess Cu from the grain.

Because Cu is both essential and toxic depending on concentration, organisms including plants have developed a finely tuned homoeostatic network to control cellular Cu concentrations. Cu homoeostasis in plants depends on the control of root uptake, root-to-shoot translocation, vacuolar compartmentation and distribution/redistribution of Cu to various organs. In plants, Cu is mainly taken up in roots by CTR-like high-affinity Cu transporters (COPT) such as COPT1 in *Arabidopsis thaliana*^10^ and rice^11^. Several heavy metal P-type ATPases have been shown to function in Cu homoeostasis in *Arabidopsis* and rice. AtHMA5 is involved in loading Cu into the xylem for root-to-shoot translocation and/or Cu detoxification in roots^12^,^13^. AtHMA6/PAA1 and AtHMA8/PAA2 are responsible for transporting Cu into chloroplasts. AtHMA6/PAA1 transports Cu across the chloroplast envelope, while the thylakoid membrane localized AtHMA8/PAA2 likely transports Cu into the thylakoid lumen^14^,^15^. AtHMA7/RAN1 has been proposed to deliver Cu to ethylene receptors^16^,^17^. In rice, OsHMA5 has been shown to be involved in loading Cu to the xylem for root-to-shoot translocation^18^. A yellow stripe-like protein, YSL16, is required for recycling Cu from older tissues to the young developing tissues as well as grains^19^. However, many transporters involved in other Cu transport processes remain unidentified.

We previously identified 134 quantitative trait loci (QTL) that control variation in the concentration of 16 elements (P, Mg, K, S, Ca, As, Cd, Co, Cu, Fe, Mn, Mo, Ni, Rb, Sr and Zn) in unmilled rice grain using two synthetic rice mapping populations^20^. On the basis of a recombinant inbred population derived from a cross between Lemont (LM, *japonica*) and TeQing (TQ, *indica*; LT-RILs), and a TeQing-into-Lemont backcross introgression lines (TILs) population, 12 QTLs controlling grain Cu concentration were identified in either one or both mapping populations under flooded and unflooded field conditions^20^. Among them a major QTL for grain Cu was detected on chromosome 2 (designated *qGCu2-1*), which explains up to 43% of variation in grain Cu in the LT-RILs. In this study, we identify the gene most likely responsible for this QTL. We find that *qGCu2-1* likely encodes a heavy metal P_1B_-type ATPase, OsHMA4. OsHMA4 localizes to the vacuolar membrane of root cells and we provide evidence that it functions in sequestering Cu into the vacuoles. Loss-of-function of *OsHMA4* results in increased root-to-shoot translocation of Cu, and subsequently increases Cu accumulation in rice grain. Furthermore, we provide evidence that the genotypic difference in grain Cu results from different transport activities of OsHMA4 for Cu due to a single amino acid substitution.

## Results

### Map-based cloning of *qGCu2-1*

**Figure 1:**
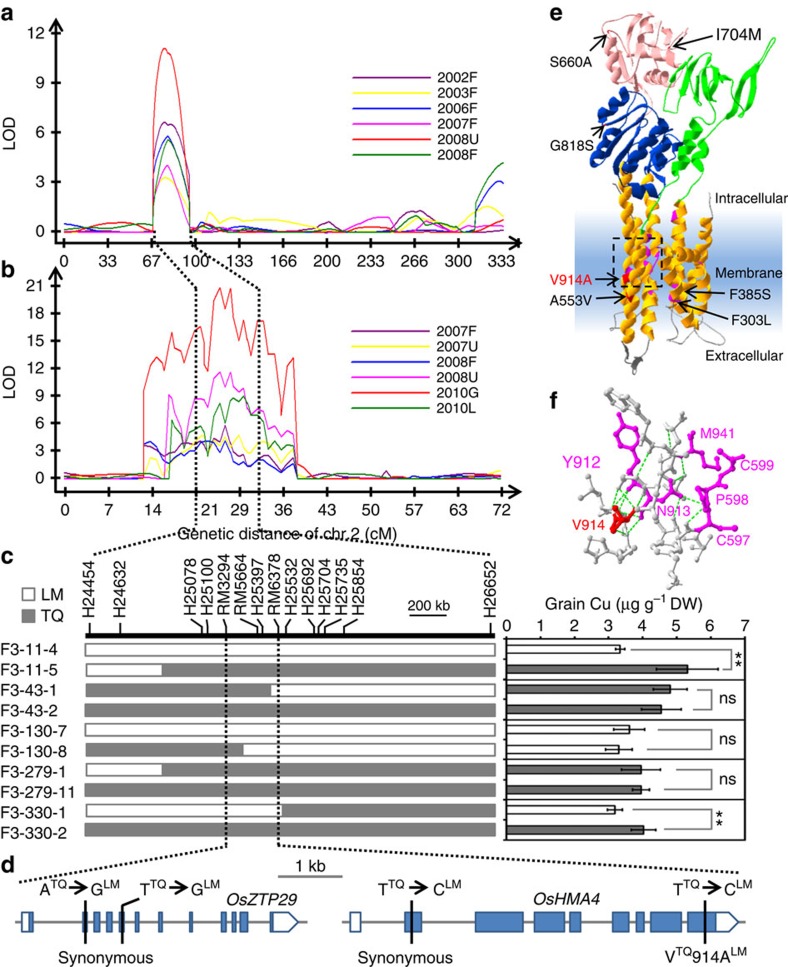
QTL analysis and map-based cloning of *qGCu2-1*. (**a**,**b**) The LOD profiling of *qGCu2-1* on chromosome 2 in the LT-RIL population (**a**) and TIL population (**b**) grown in multiple years under different conditions. F, flooded; U, unflooded. G, grains of TILs grown in greenhouse; L, leaves of greenhouse grown TILs. (**c**) Fine mapping of *qGCu2-1*. The grain Cu of homozygous F_3_ plants of five recombinants was determined. Data are presented as mean±s.d. with six individual plants. **P≤0.01 indicates significant difference (Student's *t*-test). NS, no significant difference. (**d**) Gene structure and sequence variation of two candidate genes *OsZTP29* and *OsHMA4* between TQ and LM. (**e**) Three-dimensional structural model of OsHMA4 generated by homology modelling using a *Legionella pneumophila* P-type ATPase CopA as a template. The actuator domain, phosphorylation domain, nucleotide binding domain and the transmembrane domain are shown in green, blue, red and orange, respectively. The membranous Cu binding residues are shown in magenta. Polymorphic amino acids from different varieties are shown and the polymorphic V914A between TQ and LM is highlighted in red. (**f**) Close-up structure of the region with polymorphic V914A. The YN and CPC motif are shown in magenta. The green dash lines indicate H-bonds.

To confirm the *qGCu2-1* QTL for grain Cu accumulation we detected previously^20^, we grew the LT-RIL and TIL populations over multiple years under both flooded and unflooded field conditions. This Cu QTL was consistently detected in grain of both the LT-RILs and TILs, irrespective of year or growth conditions (Fig. 1a,b). Furthermore, *qGCu2-1* was also detected in both grain and leaf tissue from TILs cultivated in the greenhouse (Fig. 1b). The reproduction of the *qGCu2-1* Cu QTL in greenhouse-cultivated material enabled us to fine map the QTL using greenhouse cultivated plants (Fig. 1b).

To fine map the *qGCu2-1* locus, we crossed four LT-RIL lines containing the chromosome fragment from TQ in the mapping region with LM and generated F_2_ progeny by self-pollination. Five plants with recombinations between markers H24454 and H26652 were isolated from 1,258 F_2_ plants. These selected F_2_ plants were self-pollinated and integration of grain Cu concentration and genotypic data of F_2:3_ progeny families narrowed the QTL interval down to a 273 kb region between the markers RM3294 and RM6378 (Fig. 1c). Among 41 genes in this region (Supplementary Table 1), 2 genes encoding putative heavy metal transporters were identified as candidate genes: a metal cation transporter gene (LOC_Os02g10230, *OsZTP29*) and a Cu-transporting ATPase gene (LOC_Os02g10290, *OsHMA4*). OsZTP29 shares 80% amino acid identity with the zinc transporter ZTP29 in *Arabidopsis*^21^. Sequence analysis revealed two synonymous single-nucleotide polymorphisms (SNPs) in the coding sequence of *OsZTP29* between the LM and TQ cultivars (Fig. 1d). Five SNPs and five small insertions and deletions were found in the promoter sequence of *OsZTP29*, which did not change its expression level between TQ and LM (Supplementary Fig. 1a), suggesting that *OsZTP29* is likely not the casual gene for *qGCu2-1*. However, comparison of *OsHMA4* sequences identified two SNPs in the coding region of *OsHMA4* between TQ and LM. Among the two SNPs, only one SNP altered the amino acid sequence, with a valine (V) in TQ and an alanine (A) in LM at amino acid 914 (Fig. 1d). Structure prediction showed that OsHMA4 has eight transmembrane domains and the polymorphic V914A occurs in the seventh transmembrane domain. Homology modelling indicates that V914A localizes in the membranous Cu-binding site I, which includes the conserved YN and CPC motifs^22^ (Fig. 1e,f; Supplementary Fig. 2). A single SNP in the 3 kb promoter region was also found between LM and TQ, but this did not alter the expression level of *OsHMA4* (Supplementary Fig. 1b) and there is no known *cis* element at this SNP.

**Figure 2:**
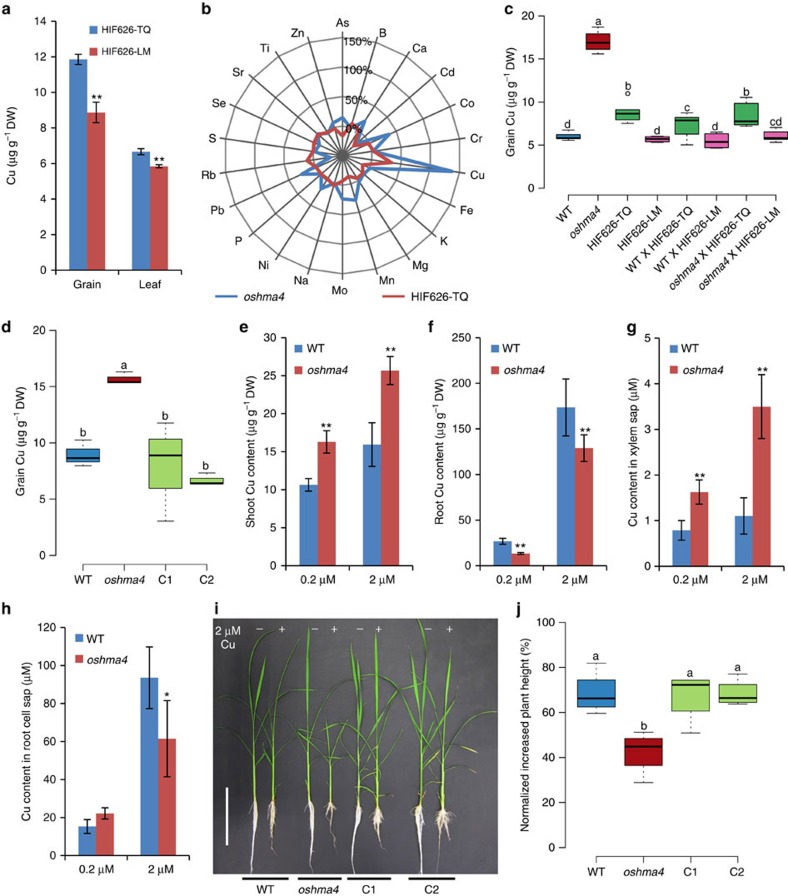
Characterization of the *oshma4* mutant and complementation test. (**a**) The Cu concentration in the grain and leaf of HIF626-TQ and HIF626-LM. (**b**) Percentage difference of 22 elements in the grain of *oshma4* compared with the WT, and HIF626(TQ) compared with HIF626(LM). Data are visualized in the radar chart. (**c**) Genetic complementation of *oshma4* by crossing with HIF626-TQ or HIF626-LM. The Cu concentration in the grain of F1 plants was determined. (**d**) Transgenic complementation of *oshma4* by transforming Nipponbare cDNA into the *oshma4* mutant. Two independent complemented lines are shown. (**e**–**h**) The Cu concentration in the shoots (**e**) and roots (**f**), xylem sap (**g**) and root cell sap (**h**). Plants were hydroponically grown in nutrient solution for two weeks and treated with 0.2 or 2 μM CuSO_4_ for another week. (**i**) The image of WT, *oshma4* and two complemented lines grown in nutrient solution without (−) or with (+) 2 μM CuSO_4_ for 15 days. Bar, 15 cm. (**j**) The relative shoot growth of plants exposed to 2 μM CuSO_4_ for 15 days. Data in **a**,**c**–**f**,**j** are presented as means±s.d. (*n*=6 in **a**,**g,** 4–8 in **c**, 3 in **d**, 12 in **e**,**f** and 4 in **h**,**j**). *P≤0.05 and **P≤0.01 indicate significant difference between WT and *oshma4* mutant, respectively (Student's *t*-test). Boxes with different letters in **c**,**d**,**j** indicate significant difference at *P*≤0.05 (Fisher's least significant difference (LSD) test). DW, dry weight.

Taking advantage of the existence of residual heterozygosity in TILs, we developed appropriate near isogenic lines (NIL) by generating heterogeneous inbred families (HIFs) (Supplementary Fig. 3)^23^,^24^. TIL626 was identified to be heterozygous at the *OsHMA4* locus. The HIF626-TQ and HIF626-LM lines were isolated in the next generation. These lines are identical at the majority of loci in the genome and only differ in a small genomic region containing homozygous *OsHMA4* alleles from TQ or LM, respectively (Supplementary Fig. 3). Elemental analysis showed that HIF626-TQ accumulated significantly higher concentrations of Cu in both grain and leaf than HIF626-LM (Fig. 2a), suggesting that the TQ allele contributes to high Cu in the grain. This is consistent with the prior QTL mapping results, where TQ was found to contribute the *qGCu2-1* allele for higher grain Cu^20^.

### Phenotypic analysis of *oshma4* knockout mutant

To test whether *OsHMA4* has a biological role consistent with being the causal gene for *qGCu2-1*, we obtained a T-DNA insertion mutant of *OsHMA4*. The T-DNA insertion in the fifth intron of *OsHMA4* completely interrupts its expression (Supplementary Fig. 4a–c). The T-DNA *oshma4* mutant is slightly shorter and has lower fertility compared to the wild-type (WT; Supplementary Fig. 5a–f). Elemental analysis showed that the Cu concentration in the grain of *oshma4* was 138% higher than that of the WT (*P*<0.001, Student's *t*-test, *n*=12) (Fig. 2b). Of the 22 elements measured, Cu is the only element that showed a major change in concentration in the grain of *oshma4*, suggesting a specific effect of *OsHMA4* on Cu (Fig. 2b). We also only observed a significant difference in grain Cu concentration between HIF626-TQ and HIF626-LM (Fig. 2b).

To further assess whether *OsHMA4* may be the causal gene for *qGCu2-1*, we crossed *oshma4* and WT with HIF626-TQ and HIF626-LM, respectively. The Cu concentration in grain of *oshma4* × HIF626-TQ F1 and *oshma4* × HIF626-LM F1 plants were significantly lower than that of the *oshma4* mutant (Fig. 2c), similar to the level in the grains of HIF626-TQ and HIF626-LM, respectively, suggesting that both the TQ and LM *OsHMA4* alleles are functional. However, a significant difference in grain Cu concentration between *oshma4* × HIF626-TQ F1 and *oshma4* × HIF626-LM F1 was observed (Fig. 2c), indicating different functional activity of the TQ and LM *OsHMA4* alleles. Furthermore, transfer of a WT DNA fragment containing the *OsHMA4* promoter region and the entire open reading frame (ORF) into the *oshma4* knockout mutant totally suppressed its high grain Cu phenotype (Fig. 2d; Supplementary Fig. 4d). These results demonstrate that *OsHMA4* is indeed responsible for the high grain Cu phenotype found in *oshma4* knockout mutant and both OsHMA4 alleles are functional, although their transport activity is different.

The high Cu phenotype of the *oshma4* mutant was observed not only in the grain but also in the blade and sheath of the flag leaf, as well as the upper nodes and internodes of the main tiller at the harvesting stage (Supplementary Fig. 5g,h). These results indicate that *OsHMA4* may not specifically control Cu in the grain but in all above ground tissues. Further analysis of seedlings grown in nutrient media with either normal or elevated Cu concentrations showed that *oshma4* plants had significantly lower Cu concentrations in the roots and higher Cu concentrations in the shoots compared with the WT (Fig. 2e,f). However, there was no difference in other metal concentrations, including Cd, Ag, Co and Pb (Supplementary Fig. 6a–e). Cu concentration in the xylem sap was also higher in *oshma4* than in the WT (Fig. 2g). Furthermore, the Cu concentration in the root cell sap, which is mainly composed of the contents of the vacuole^25^, was significantly lower in the *oshma4* mutant compared to the WT (Fig. 2h).

We also compared Cu tolerance between the *oshma4* mutant and WT. The *oshma4* mutant was more sensitive to elevated Cu concentrations in the growth media compared with the WT (Fig. 2i,j; Supplementary Fig. 5i). Furthermore, such increased Cu sensitivity of *oshma4* was rescued in the transgenic complementation lines (Fig. 2i,j).

### Expression pattern and subcellular localization of OsHMA4

**Figure 3:**
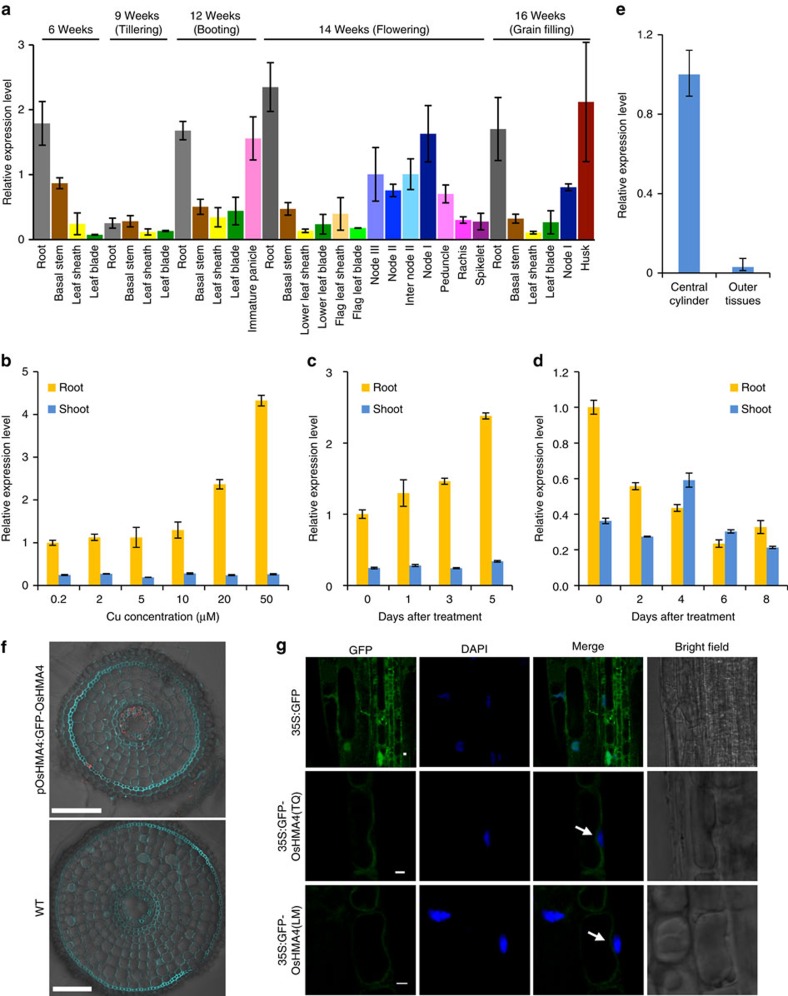
Expression pattern and subcellular localization of OsHMA4. (**a**) Expression level of *OsHMA4* in different organs at different growth stages. Samples were taken from Nipponbare grown in a paddy field. (**b**) Expression of *OsHMA4* in TQ grown in nutrient solution with different concentrations of Cu for two weeks. (**c**) Time-course expression of *OsHMA4* under Cu treatment. Two-week-old TQ plants were treated with 20 μM Cu for indicated days. (**d**) Expression of *OsHMA4* was suppressed under Cu deficiency. TQ plants were grown with 0.2 μM CuSO_4_ for one week and treated with Cu free nutrient solution for indicated days. Expression level of *OsHMA4* was quantified by qRT-PCR. Data were presented as mean±s.d. (*n*=3). (**e**) *OsHMA4* mainly expresses in central cylinder tissue in roots. Tissues were separated by laser microdissection for qRT-PCR. (**f**) Tissue-specific expression of *OsHMA4* in roots. Immunostaining using an antibody against GFP was performed in the root of WT (lower) or transgenic rice expressing *GFP-OsHMA4* driven by *OsHMA4* native promoter in *oshma4* (upper). Red colour indicates the GFP-specific signal. Blue colour indicates cell wall and nucleus stained by 4,6-diamidino-2-phenylindole (DAPI). Bar, 100 μm. (**g**) Subcellular localization of OsHMA4 in stable *A. thaliana* transgenic plants. The nuclei were stained by DAPI. The arrows indicate the vacuolar membrane on the inner side of the nucleus. Bar, 2 μm.

Expression of *OsHMA4* was observed in most organs throughout the growth period of Nipponbare grown in a paddy field (Fig. 3a). However, the expression of *OsHMA4* was generally much higher in the roots compared with other organs (Fig. 3a). Similar expression pattern was observed in TQ grown in a greenhouse (Supplementary Fig. 7a). Expression of *OsHMA4* was strongly induced by high Cu treatment in the roots but not in the shoots (Fig. 3b,c). *OsHMA4* was also slightly induced by Ag and Cd treatment but suppressed by Pb and Mn treatment (Supplementary Fig. 7b). Under Cu-depleted growth conditions, expression of *OsHMA4* was downregulated in the root (Fig. 3d). Such downregulation was also observed under Fe depletion but not under Zn or Mn depletion (Supplementary Fig. 7c). The induction by excess Cu and downregulation by Cu deficiency suggested a critical role for *OsHMA4* in Cu homoeostasis in roots.

Using laser microdissection, we observed that *OsHMA4* was mainly expressed in the central cylinder of the mature root (Fig. 3e). To further investigate the tissue-specific localization of *OsHMA4*, we expressed *GFP-OsHMA4* in the *OsHMA4* mutant under the control of the native promoter of *OsHMA4* (Supplementary Fig. 5j). Immunostaining with an anti-green fluorescent protein (GFP) antibody revealed OsHMA4 to accumulate in the vascular tissues of the stele, mainly in pericycle cells (Fig. 3f). This observation was further supported by β-glucuronidase (GUS) staining of the *OsHMA4* promoter-GUS transgenic rice lines (Supplementary Fig. 7d–f).

Analysis of the subcellular localization of GFP-OsHMA4 after stable heterologous expression in *Arabidopsis* showed OsHMA4 to be localized to the tonoplast (Fig. 3g). We observed that OsHMA4 from both TQ and LM were localized to the tonoplast, suggesting that the V914A variation has no effect on the subcellular localization of OsHMA4 (Fig. 3g). Subcellular localization of OsHMA4 was further investigated in transgenic rice expressing *GFP-OsHMA4* under the control of the native promoter of *OsHMA4*. Western blot analysis with a GFP antibody showed a single band with the predicted size in the rice transgenic line, but not in WT, indicating the specificity of the GFP antibody (Supplementary Fig. 8a). *In situ* immunostaining with this GFP antibody further showed that at least part of the signal was localized to the tonoplast, with the immunostaining being observed on the inside facing side of the nuclei (Supplementary Fig. 8c–e). We further showed that the tonoplast localization of OsHMA4 is unlikely to be affected by excess Cu (Supplementary Fig. 8f). Immunoblotting with GFP antibody of sucrose-density gradient separated microsomal membranes from *GFP-OsHMA4* expressing rice roots revealed a weak signal for GFP-OsHMA4 in the tonoplast and plasma membrane (Supplementary Fig. 8b).

### Heterologous expression in *Arabidopsis* and yeast

**Figure 4:**
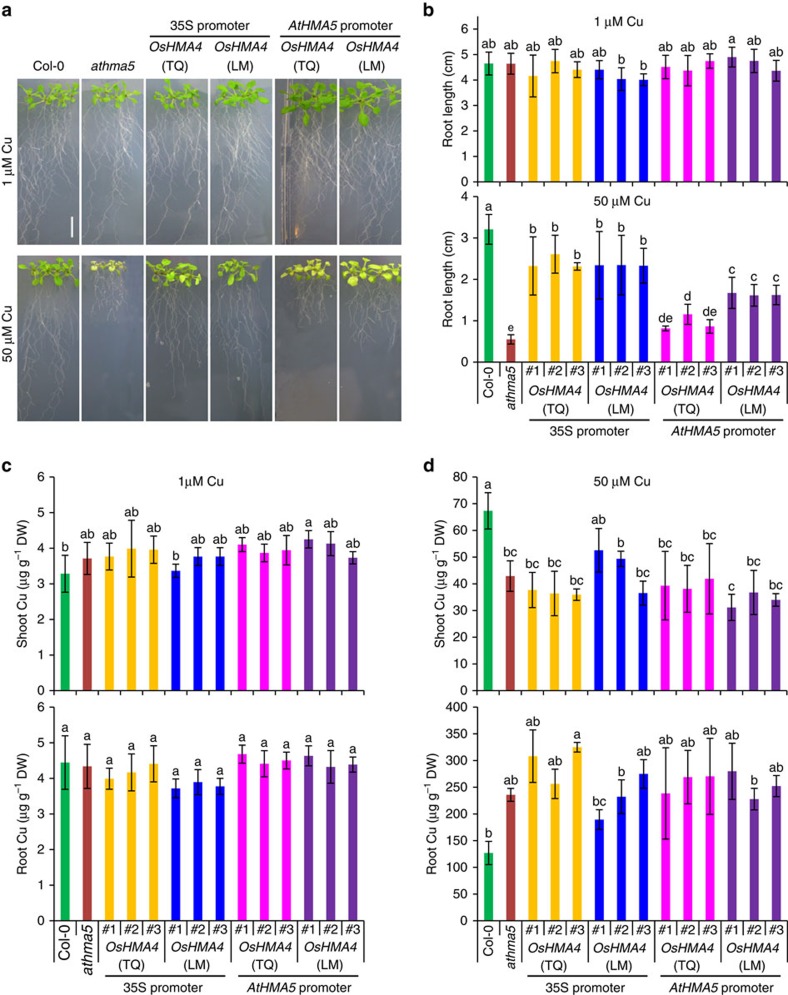
Expression of *OsHMA4* in *Arabidopsis athma5* mutant alleviates its Cu sensitive phenotype. (**a**) Phenotype of *athma5* mutant transformed with *OsHMA4* from TeQing (TQ) or Lemont (LM) driven by CaMV 35S promoter or *AtHMA5* native promoter. Plants were grown on MGRL media with 1 μM CuSO_4_ for 6 days and then transferred to the media containing 1 or 50 μM CuSO_4_ for 10 days. Bar, 1 cm. (**b**) Root length of *OsHMA4* transgenic lines grown on the media containing 1 or 50 μM CuSO_4_. Plants were grown on MGRL media with 1 μM CuSO_4_ for 3 days and then transferred to the media containing 1 or 50 μM CuSO_4_ for 7 days. Three independent lines for each construct were shown. (**c**,**d**) Cu concentration in the shoots and roots of *OsHMA4* transgenic lines grown on the media containing 1 μM (**c**) or 50 μM CuSO_4_ (**d**). Plants were grown as in (**a**) and the same lines as in **b** were used. Cu concentration was determined by ICP-MS. Data in **b**–**d** were shown as means±s.d. with *n*=9 to 16 in **b** and *n*=3 in **c** and **d**. Columns with different letters in **b**–**d** indicate significant difference (*P*≤0.01, Fisher's LSD test). DW, dry weight.

Sequence analysis revealed that OsHMA4 shares 56.5% sequence identity with *Arabidopsis* AtHMA5. AtHMA5 has been shown to be involved in Cu translocation from roots to shoots and/or Cu detoxification in roots^12^,^13^. To investigate whether expression of *OsHMA4* in *athma5* could improve its tolerance to excess Cu, we heterologously expressed *GFP-OsHMA4* from TQ and LM in *athma5* using the 35S promoter (Supplementary Fig. 9a). When grown in low Cu, the root elongation of *athma5* knockout mutant was similar to that of the WT and transgenic lines carrying *GFP-OsHMA4* from either TQ or LM in the *athma5* background (Fig. 4a,b; Supplementary Fig. 10a,b). However, under high Cu, the root elongation of the *athma5* mutant was significantly inhibited, but the introduction of *OsHMA4* from either TQ or LM into the *athma5* mutant significantly increased its Cu tolerance (Fig. 4a,b; Supplementary Fig. 10a,b). Quantification of Cu showed that the Cu concentration in *athma5* was lower in the shoots and higher in the roots compared with the WT when grown with 50 μM Cu. However, in the transgenic lines expressing either allele of *OsHMA4*, Cu levels in both roots and shoots were similar to that of the *athma5* mutant (Fig. 4c,d). These results suggest that although *OsHMA4* can suppress the Cu sensitivity of *athma5* this is not achieved by restoring root-to-shoot translocation of Cu, but rather most likely by enhancing sequestration of Cu into root vacuoles. To rule out the ectopic effect of overexpression of *OsHMA4* driven by 35S promoter, we also expressed *OsHMA4* in *athma5* using the *AtHMA5* native promoter. Expression of the LM *OsHMA4* allele from the *AtHMA5* native promoter significantly enhanced the resistance of *athma5* to excess Cu, whereas the TQ allele had no consistent affect (Fig. 4a,b; Supplementary Fig. 9b; Supplementary Fig. 10a,b). These results suggest that the TQ *OsHMA4* allele is hypofunctional relative to the LM allele.

To investigate whether expression of *OsHMA4* in WT *Arabidopsis* could improve its tolerance to excess Cu, we heterologously expressed *GFP-OsHMA4* in Col-0 WT using the 35S promoter (Supplementary Fig. 9c). These transgenic lines were more tolerant to Cu stress compared with non-transgenic WT (Supplementary Fig. 11a–d). Quantification of Cu showed that under excess Cu, the *GFP-OsHMA4* expressing lines accumulated more Cu in roots than that of WT, further supporting a role for OsHMA4 in sequestration of Cu into root vacuoles (Supplementary Fig. 11e,f). The expression of Cu deficiency responsive genes was not markedly affected in the *Arabidopsis* lines expressing *GFP-OsHMA4* with the exception of *ZIP2*, which was induced in shoots of some lines (Supplementary Fig. 9d).

**Figure 5:**
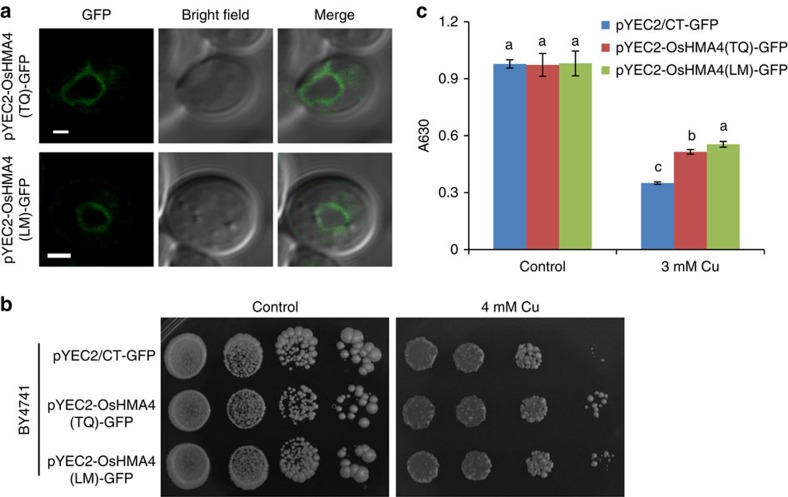
Functional analysis of OsHMA4 in yeast. (**a**) Subcellular localization of OsHMA4 in yeast. *OsHMA4* from TeQing (*OsHMA4(TQ)*) or from Lemont (*OsHMA4(LM)*) were expressed in a centromeric plasmid pYEC2/CT–GFP. GFP signals were observed mainly on the tonoplast. Bar, 1 μm. (**b**) Expression of *OsHMA4* in yeast using a centrimeric plasmid pYEC2/CT–GFP enhances Cu tolerance. Overnight yeast cell suspension of BY4741 transformed with empty vector pYEC2/CT–GFP or *OsHMA4* from TQ or LM were serially diluted (1:10) and spotted on the media without (Control) or with 4 mM CuSO_4_. Pictures were taken after 5 days growth at 30 °C. (**c**) The absorbance at 630 nm (A630) of cell cultures of BY4741 transformed with the empty vector pYEC2/CT–GFP or *OsHMA4-GFP* from TQ or LM. Yeast strains were grown in liquid media containing without (Control) or with 3 mM CuSO_4_ for 24 h. Data were shown as means±s.d. with three independent colonies. Columns with different italic or non-italic letters indicate significant difference under control or 3 mM CuSO_4_, respectively (*P*≤0.01, Fisher's LSD test).

To characterize the Cu transport activity of OsHMA4, we expressed *OsHMA4* in the yeast WT strain BY4741 using a low copy number centromeric plasmid pYEC2/CT–GFP. GFP signals were specifically observed at the vacuolar membrane of yeast transformed with either *pYEC2-OsHMA4(TQ)–GFP* or *pYEC2-OsHMA4(LM)–GFP* (Fig. 5a), supporting the tonoplast localization of OsHMA4 we observed in both rice and *Arabidopsis*. Yeast expressing *OsHMA4-GFP* were more tolerant to Cu stress than those transformed with the empty vector (Fig. 5c,d), supporting a function for OsHMA4 in transport of Cu into yeast vacuoles. Expression of the cucumber tonoplast-localized CsHMA5.1 and CsHMA5.2 using a similar low copy number centromeric plasmid has also been shown to improve Cu tolerance in yeast^26^.

To further test the Cu transport activity of OsHMA4, we expressed *OsHMA4* in the yeast *ccc2* mutant using a high copy number 2μ origin plasmid pYES2. The Cu-transporting P-type ATPase CCC2 localizes at the late- or post-Golgi compartment and delivers Cu to the multi-copper oxidase Fet3P, which is required for high-affinity Fe uptake at the plasma membrane^27^. The *ccc2* knockout mutant is unable to grow on Fe-deficient media (Supplementary Fig. 12a). We observed that expression of *OsHMA4* from either TQ or LM in the *ccc2* yeast mutant was able to restore growth of the yeast mutant on Fe-deficient media, suggesting that OsHMA4 has Cu transport activity in yeast (Supplementary Fig. 12a). Analysis of the GFP signal in yeast expressing *OsHMA4-GFP* from the high copy number 2μ origin pYES2 plasmid revealed that OsHMA4-GFP localizes throughout the endomembrane system, likely including the late- or post-Golgi compartment (Supplementary Fig. 12f). Such mislocalization of OsHMA4-GFP to the late- or post-Golgi compartment when accumulated to high levels explains how *OsHMA4* can complement the *ccc2* growth defect when expressed from pYES2. Supporting this conclusion that OsHMA4 can transport Cu in yeast, we also observed that expressing *OsHMA4* in either WT yeast or a yeast strain lacking the high-affinity Cu transporter *CTR1* increased their sensitivity to excess Cu (Supplementary Fig. 13a). Furthermore, expression of *OsHMA4* did not alter the sensitivity of yeast to other heavy metals such as Cd, Co, Ag, Pb, Mn and Zn (Supplementary Fig. 13b), suggesting that OsHMA4 transports Cu specifically. Significantly, WT yeast expressing the LM *OsHMA4* allele was more sensitive to excess Cu than WT yeast expressing the TQ *OsHMA4* allele (Supplementary Fig. 12b–e), suggesting stronger Cu transport activity of OsHMA4 from LM. The increased sensitivity to Cu of yeast expressing OsHMA4 from pYES2 can again be explained by the mislocalization of OsHMA4 to internal membrane compartments that are sensitive to enhanced Cu transport. This is similar to previous studies in which expression of the normally tonoplast-localized OsHMA3 or AtHMA3 in yeast using the high copy number plasmid pYES2 increases the sensitivity to Cd stress rather than enhancing Cd tolerance^28^.

### Analysis of genetic variation of *OsHMA4*

**Figure 6:**
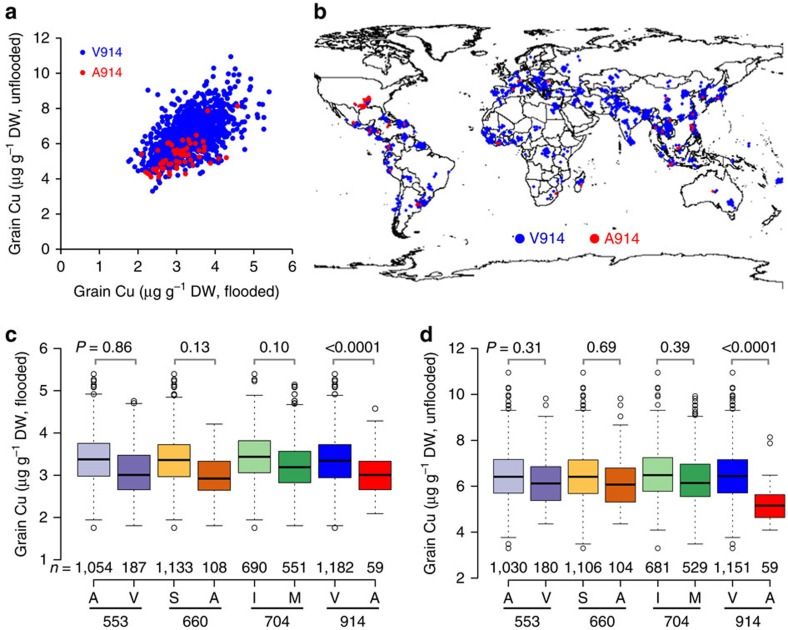
Natural allelic variation at the *OsHMA4* loci underlies the variation of grain Cu in rice. (**a**) The grain Cu of USDA core collection rice accessions with the strong (A914) and weak allele (V914) of OsHMA4 when grown under flooded or unflooded condition. (**b**) Distribution of rice accessions with the strong and weak allele of OsHMA4. (**c**,**d**) The grain Cu of rice accessions with different OsHMA4 haplotypes grown under flooded (**c**) or unflooded condition (**d**). Data are shown as boxplots. Numbers under the boxes are the accession number; numbers above the boxes are the *P* values generated by the generalized least squares model. DW, dry weight. The map image was generated using R package rworldmap^64^ with data derived from Natural Earth v1.4.0 (http://www.naturalearthdata.com/).

To gain insight into the contribution of genetic variation at *OsHMA4* to variation in grain Cu across the *O. sativa* species, we analysed the sequence of *OsHMA4* in the genomes of 950 diverse worldwide rice accessions^29^. This analysis revealed nine non-synonymous polymorphisms in the coding sequence of *OsHMA4*, including the polymorphic V914A we discovered between TQ and LM (Supplementary Table 2; Supplementary Fig. 2; Fig. 1e). The allele frequency of the strong allele of *OsHMA4* from LM (A914) was very low (0.0068), suggesting that it is a rare allele (Supplementary Table 2). To further associate genetic variation at *OsHMA4* with rice grain Cu, we genotyped *OsHMA4* in 1,349 worldwide accessions from the USDA Rice Core Collection for which we had previously reported the concentration of grain Cu^30^. We identified five of the previous nine non-synonymous polymorphisms and also identified 67 accessions with the LM-like strong allele (5.1%; Supplementary Table 2). The accessions with the strong *OsHMA4* allele (A914) generally accumulate less grain Cu and are largely distributed in the USA (26 of 67; Fig. 6a,b). When taking the kinship between the accessions into account, we only observed a significant difference in grain Cu between the two alleles polymorphic at the V914A site, but not at the other three polymorphic sites that have a minor allele frequency higher than 0.05 (Fig. 6c,d). These polymorphisms explain a significant amount of the variation in grain Cu of this diverse core collection when grown in either flooded (8.3%; F_4, 1,236_=28, *P*<0.0001, generalized least squares approach, *n*=1,182/59) or unflooded conditions (8.6%; F_4, 1,205_=28.55, *P*<0.0001, generalized least squares approach, *n*=1,151/59). One likely source of the strong allele we first identified in LM is its ancestor Fortuna, which was selected from a landrace from Taiwan, suggesting that the strong allele may originate from Asia (Supplementary Fig.14a). This strong allele of *OsHMA4* was not found in 446 accessions of wild rice *Oryza rufipogon*, the immediate ancestral progenitor of Asian cultivated rice *Oryza sativa*, or in African rice *Oryza glaberrima* (20 accessions) and its progenitor *Oryza barthii* (94 accessions)^31^,^32^. Grain Cu is generally lower when plants are grown in flooded compared with unflooded paddy conditions (Fig. 6c,d), which is likely due to lower Cu bioavailability in water-logged soil. The reasons for this reduction in Cu bioavailability under flooded conditions are complex and involve a decrease in redox potential^33^.

## Discussion

To cope with the dual nature of Cu being essential for cells and toxic when present in excess, plants have evolved sophisticated mechanisms to control the cellular Cu concentration. One strategy is to sequester excess Cu into vacuoles. This vacuolar Cu serves as a reservoir for Cu that can be remobilized under Cu deficiency. However, the molecular mechanism underlying this process in plants is poorly understood. In this study, we identified a QTL controlling rice grain Cu and determined the likely causal gene to be *OsHMA4*. *OsHMA4* belongs to the Cu^+^/Ag^+^ subgroup of HMA and we provide evidence it encodes a tonoplast-localized transporter specific for Cu. We found that the genotypic difference in grain Cu was not due to the expression level or subcellular localization of *OsHMA4*, but most likely the transport activity for Cu (Supplementary Fig. 1b; Fig. 3g; Supplementary Fig. 8e; Supplementary Fig. 12b–e). OsHMA4 from the low Cu cultivar (LM) showed stronger transport activity for Cu than that from the high Cu cultivar (TQ) when expressed in both *Arabidopsis* and yeast (Figs 4a,b and 5c; Supplementary Fig. 12b–e). Knockout of *OsHMA4* resulted in increased Cu concentration in the shoots and xylem sap, but decreased Cu in the roots and root cell sap (Fig. 2e–h). We conclude that OsHMA4 most likely functions to sequester Cu into vacuoles of pericycle cells to help control the root-to-shoot translocation of Cu. This likely vacuolar sequestration of Cu by OsHMA4 is also associated with enhanced Cu tolerance (Fig. 2i,j; Supplementary Fig. 5i).

Several transporters have been shown to compartmentalize various heavy metals in root vacuoles. For example, *Arabidopsis* AtHMA3 participates in vacuolar sequestration of Cd, Zn, Co and Pb^34^,^35^, and AtMTP3 is involved in sequestration of Zn^36^. Recently, two cucumber P_1B_-type ATPases CsHMA5.1 and CsHMA5.2 have been shown to transport Cu into vacuoles in yeast^26^. However, their *in vivo* functions in regulating Cu homoeostasis remains unclear. In *Arabidopsis*, the protein mediating the transport of Cu into vacuoles has not been identified. OsHMA5 from rice and AtHMA5 from *Arabidopsis* are involved in loading Cu into the xylem for root-to-shoot translocation and/or Cu detoxification in roots^12^,^13^,^18^. Several other HMA proteins in *Arabidopsis* have been shown to have Cu-transporting activity but none of them are involved in the efflux of Cu into vacuoles. For example, AtHMA6/PAA1 and AtHMA8/PAA2 are required for Cu transport in chloroplasts and AtHMA7/RAN1 delivers Cu to ethylene receptors on the Golgi membrane^14^,^15^,^16^,^17^. Even though the functional counterpart of OsHMA4 is likely absent in *Arabidopsis*, several OsHMA4 homologues have been identified in other species such as sorghum, poplar and grape vine^26^.

Expression of *OsHMA4* was not induced by short-term Cu treatment in a previous study^37^. However, here we demonstrated that *OsHMA4* is induced under longer-term exposure to excess Cu (Fig. 3b,c). This is consistent with the proposed function of *OsHMA4* in compartmentalization of Cu into vacuoles after exposure to excess Cu. Furthermore, the expression of *OsHMA4* is suppressed by Cu deficiency (Fig. 3d). Such downregulation under Cu deficiency may decrease Cu sequestration in the vacuolar storage pool.

OsHMA4 is characteristically localized at the pericyle cells of root mature zones (Fig. 3e). This localization is similar to OsHMA5, a plasma membrane-localized Cu transporter responsible for xylem loading of Cu^18^. This suggests that OsHMA4 plays a role in fine tuning cellular Cu concentration before loading to the xylem depending on Cu concentration in the environment. This localization is also different from other tonoplast-localized transporter in rice roots such as OsHMA3, which is localized in all root cells^28^. Furthermore, OsHMA4 is also characterized by its transport specificity for Cu (Supplementary Fig. 6a–e; Supplementary Fig. 13b). Among the HMA members characterized in rice, OsHMA2 and OsHMA3 transport both Cd and Zn^38^,^39^,^40^,^41^, while OsHMA9 transports Cu, Zn, Pb and Cd^37^. The mechanisms underlying the transport substrate specificity remain to be examined.

The strong allele of OsHMA4 from LM results from a single amino acid change at position 914 (from V to A; Fig. 1d). This amino acid is localized in the seventh transmembrane domain (Fig. 1e,f; Supplementary Fig. 2), which is predicated to be the Cu-binding site I including the conserved YN and CPC motifs^22^ (Fig. 1e,f; Supplementary Fig. 2). Thus, the V914A variant might differ in Cu binding activity and thus have different transporting activity. The absence of the strong allele of *OsHMA4* (A914) in wild rice *O. rufipogon*, the immediate ancestral progenitor of Asian cultivated rice *O. sativa*, suggested that the weak *OsHMA4* allele (V914) is ancestral, and the strong allele might have arisen during the domestication of *O. sativa*^29^. However, the lack of evidence for a selective sweep of the genome around *OsHMA4* suggests that this strong allele of *OsHMA4* was not selected during domestication^29^. The strong allele of OsHMA4 is rare in the worldwide rice population perhaps explaining why this functional polymorphism was not identified in a recent genome-wide association study^42^.

In summary, we have identified *OsHMA4* as the most likely causal gene underlying the QTL for Cu accumulation in rice grain through multiple year field and greenhouse trials. OsHMA4 localizes to the vacuolar membrane and we provide evidence that it functions to transport Cu into vacuoles in roots. The identification of natural variation at the *OsHMA4* loci associated with rice grain Cu provides an efficient way to breed rice varieties with Cu enrichment in the grain, which may be helpful in solving global Cu micronutrient deficiency. The novel *OsHMA4* alleles identified and the molecular markers developed in this work can be directly used in breeding to develop rice varieties with grain Cu concentrations that are tuned to both the concentration of Cu in the soil and the dietary needs of the population's consuming the grain.

## Methods

### Plant materials and growth conditions

The LT-RILs were derived from a cross between a US tropical *japonica* rice cultivar Lemont (LM) and a Chinese *indica* cultivar TeQing (TQ) by single-seed descent^43^,^44^. The LT-RILs contained 280 lines and were genotyped using 175 restriction fragment length polymorphism markers^44^. The LT-RILs grown in the field in 2002, 2003, 2006, 2007 and 2008 were in the F_15_, F_16_, F_17_, F_18_ and F_19_ generations, respectively. The TILs contain 123 lines and were originally genotyped using 168 simple sequence repeat (SSR) markers^45^. Plants were grown under flooded and unflooded condition in 2007 and 2008 as described previously^20^. Due to a storm, the LT-RILs grown under unflooded condition were destroyed in 2007 and thus were not included in the analysis. In 2010, the TIL population was grown in a greenhouse at Purdue University, West Lafayette, Indiana, USA. Plants were grown in 10 × 10 × 12 cm pots with sandy soil Profile Greens Grade (Profile Products, LLC, Illinois, USA) and the irrigation was controlled by an automatic system. Plants were irrigated every day with tap water to maintain the water level to 1/3 of the pot depth, and fertilized once a week with water-soluble fertilizer (15N–1.3P–13.3K; Greencare Fertilizers, Kankakee, Illinois, USA) until seeds were collected.

HIFs were generated as previously described^23^,^24^. A derived cleaved amplified polymorphic sequences (dCAPS) marker was developed based on the causal polymorphism of T4656C on *OsHMA4* between TQ and LM. TIL626 was identified as heterozygous on this dCAPS. The plants fixed with TQ allele (HIF626-TQ) and plants fixed with LM allele (HIF626-LM) were selected in self-pollinated progeny plants of TIL626.

The T-DNA insertion mutant for *OsHMA4* (PFG_1B-07418) was obtained from Kyung Hee University, Korea (http://cbi.khu.ac.kr/RISD_DB.html)^46^. The progeny plants segregated from 1B-07418 without the T-DNA insertion in the *OsHMA4* gene were used as a WT control. The mutant and WT were genotyped by PCR and expression level of *OsHMA4* in the mutant was determined by reverse transcriptase–PCR (RT-PCR; Supplementary Fig. 4b,c; see full gel images in Supplementary Fig. 15a). For analysis of the grains and different tissues of the WT and the *oshma4* mutant, plants were grown in the greenhouse. For the hydroponic experiment, WT and the *oshma4* mutant were grown as previously described^47^. Seeds of WT and *oshma4* were kept at 42 °C for at least 1 week to break any possible dormancy, soaked in water at room temperature for 2 days, and then germinated at 37 °C for 1 day. The most uniformly germinated seeds were sown in a 96-well plate from which the bottom was removed. The plate was put in a pipette tip box (12 × 8.5 × 7.5 cm) and floated in water for 1 days at 37 °C in the dark to encourage root growth. Plants were propagated in a growth chamber with a 12-h light (26 °C)/12-h dark (22 °C) photoperiod, 50–60% relative humidity and 700 μmol m^−2^ s^−1^ light. After 5 days, the seedlings were cultured with half-strength Kimura B solution^18^ and the nutrient solution was renewed every 2 days. The nutrient solution was prepared using deionized water except for the element deficiency experiment in which Milli-Q water was used. For some experiments, the WT (cv. Dongjin) and T-DNA mutant were grown hydroponically in a closed greenhouse of the Institute of Plant Science and Resources, Okayama University as described previously^18^.

### Tissue elemental analysis

The elemental concentration of the grain and other organs was determined using an inductively couple plasma mass spectrometer (ICP-MS; Elan DRCe, PerkinElmer; or NexION 300D, PerkinElmer) as described previously^20^. The grains of LT-RILs and TILs were dehulled using a modified Satake TH035A sheller (Satake Engineering, Co. Ltd., Tokyo, Japan) with the rubber liner on the rollers replaced with PU40 Polyurethane plastic to prevent the contamination of Zn^20^. Grains of WT and the *oshma4* mutant were dehulled manually. For determination of elemental concentrations in the blade, sheath, rachis, node and internode, tissues were washed with Milli-Q water and dried at 88 °C overnight. For analysis of roots from hydroponically grown plants, roots were excised from the plants, washed with 0.5 mM CaCl_2_ solution three times, rinsed with Milli-Q water once and dried at 88 °C overnight. Samples were digested with concentrated HNO_3_ at 118 °C for 4 h before ICP-MS analysis.

### QTL analysis and fine mapping of *qGCu2*

QTL analyses were performed using Windows QTL cartographer version 2.5 (http://statgen.ncsu.edu/qtlcart/WQTLCart.htm) using composite interval mapping. The composite interval mapping analysis was run using Model 6 with forward and backward stepwise regression, a window size of 10 cM, and a walk speed of 1 cM. The threshold for detection of a QTL was set at a LOD score of 3.0 following 1,000 permutations at 0.05 significant level. For fine mapping of *qGCu2-1*, four LT-RILs (LT:390, LT:528, LT:550, LT:597) with the TQ genotype at the mapping region were crossed with LM. F_2_ population was generated by self-pollination F_1_s. Two markers H24454 and H26652 were used to detect the recombinantion events that occurred around *qGCu2-1* and 12 molecular markers were developed for fine mapping. Five recombinants were isolated from 1,258 F_2_ plants and fixed recombinant F_3_ plants were generated by self-pollination. The Cu concentration in the grain and leaf of fixed recombinants was determined by ICP-MS. By progeny testing, *qGCu2-1* was fine mapped between the markers RM3294 and RM6378.

### Transgenic complementation test

For transgenic complementation experiment, *oshma4* was transformed with *OsHMA4* under the control of its own promoter. The coding region of *OsHMA4* linked with the nopaline synthase (NOS) terminator was amplified using the plasmid GFP-OsHMA4 (described below) as the template. The fragment was digested with *Bam*HI and *Bgl*II and then subcloned into the binary vector pTF101.1 (ref. 48), which was digested with *Bam*HI and dephosphorylated with Shrimp Alkaline Phosphatase (Takara). The 3,016 bp region upstream of the initiation codon of *OsHMA4* was amplified by PCR from the genomic DNA. The fragment was digested with *Spe*I and *Bam*HI and then ligated into the *Xba*I-*Bam*HI site of pTF101.1-OsHMA4. After being sequenced for confirmation (ABI PRISM 3,130 Genetic Analyzer, Applied Biosciences), the resulting plasmid *pOsHMA4:OsHMA4* was subsequently introduced into *Agrobacterium tumefaciens* (strain EHA101). Callus was induced from mature embryos of the *oshma4* mutant for Agrobacterium-mediated transformation^49^. The expression level of *OsHMA4* in transgenic plants was detected by RT-PCR. For grain Cu determination, the complementation lines, WT rice and the *oshma4* mutant were transplanted into 3.5 l plastic pots filled with paddy soil. The plants were grown in a closed greenhouse under natural light at 25–30 °C until mature. Brown rice was collected for digestion and measurement as described above. The primer sequences used are listed in Supplementary Table 3.

### Tissue specificity of *OsHMA4* expression

To investigate the tissue and cellular specificity of *OsHMA4* expression, the *GFP-OsHMA4* fusion was expressed in the background of the *oshma4* mutant under the control of its own promoter (3,016 bp). The full-length complementary DNA (cDNA) of *OsHMA4* was ligated to the 3′ end of *GFP* with the coding sequence for seven additional amino acids (SGGGGGG) digested with *Bsp*EI to generate the plasmid *GFP-OsHMA4* (ref. 37). The fused *GFP-OsHMA4* fragment was inserted between the promoter fragment and NOS terminator to produce the *pOsHMA4:GFP-OsHMA4* plasmid. This construct was introduced into the *oshma4* T-DNA knockout mutant as described above.

For further analysis of the tissue expression pattern of *OsHMA4*, the 2,630-bp promoter sequence of *OsHMA4* was PCR amplified and subcloned into the *Pst*I-*Bam*HI site of pTF101.1-GUS vector (modified from pTF101.1 (ref. 48) by insertion of the sequence of *GUS* into the *Hind*III–*Eco*RI site of pTF101.1). The resulting plasmids were transformed into rice *japonica* cv. Nipponbare. Rice transformation was performed in Iowa State University Plant Transformation Facility. GUS histochemical staining was performed as described previously^46^. The primer sequences are listed in Supplementary Table 3.

### Subcellular localization and tissue expression pattern of *OsHMA4*

To investigate the subcellular location of OsHMA4, the full-length coding sequence of *OsHMA4* was amplified from cDNA synthesized from TQ or LM using the primers listed in Supplementary Table 3, and ligated into the *Xba*I–*Bam*HI site of p1301GFP vector^47^. The resulting plasmids *35S:GFP-OsHMA4(TQ)* and *35S:GFP-OsHMA4(LM)* were transformed into *A. tumeraciens* strain GV3101 and introduced into the *Arabidopsis AtHMA5* mutant (SALK_040252) using the floral dip method^50^. The roots of T3 transgenic plants were examined using a confocal laser scanning microscope (Carl Zeiss LSM700). GFP was excited using an argon laser at 488 nm and the emission was collected between 505 and 530 nm. To visualize the nuclei, roots were incubated with 1 μg ml^−1^ of 4,6-diamidino-2-phenylindole (Molecular Probes) for 5 min at room temperature and washed five times with PBS buffer. 4,6-diamidino-2-phenylindole were excited with a ultraviolet laser at 395 nm.

### Immunostaining and western blot analysis

Immunostaining was carried out on roots of WT and transgenic rice plants expressing *GFP-OsHMA4* driven by *OsHMA4* native promoter in *oshma4* using an antibody against GFP (A-11122; Molecular Probes) as described previously^18^. For western blot analysis, the WT and transgenic lines expressed expressing *GFP-OsHMA4* driven by *OsHMA4* native promoter in *oshma4* were used. Plants were grown hydroponically for 35 days in the half-strength Kimura B solution, and then treated with 2 μM Cu for 6 h before harvesting for protein extraction. The microsome isolation and fractionation were performed according to the method described previously with slight modifications^28^. The suspended microsomes were fractionated with discontinuous sucrose gradients (20–60% sucrose in 10 mM Tris-HCl, pH 7.6, 1 mM EDTA, and 1 mM DTT) by ultracentrifugation at 100,000*g* for 18 h. The fractionated membranes were recovered by ultracentrifugation at 100,000*g* for 40 min. Each pellet was resuspended for the concentration measurement and further analysis. Equal amounts of samples were incubated at 95 °C for 2 min and then loaded into the SDS-PAGE gels (5–20% gradient polyacrylamide, ATTO, Japan). The transfer to polyvinylidene difluoride membrane was performed with a semidry blotting system, and the membrane was treated with the purified primary rabbit anti-GFP (A-11122; Molecular Probes; 20,000 times dilution) in an Immnunoreaction Enhancer Solution (Can Get Signal, TOYOBO, Japan), anti-V-ATPase (AS07213, Agrisera; 10,000 times dilution), anti-H^+^-ATPase polyclonal antibodies (AS07260, Agrisera; 10,000 times dilution), and Anti-Bip (COP-080017, Cosmo bio; 10,000 times dilution). ECL peroxidase-labelled anti-rabbit antibody (W4011, Promega; 10,000 times dilution) was used as a secondary antibody, and an ECL Plus western blotting detection system (GE Healthcare) was used for detection via chemiluminescence (Bio-Rad). The protein amount was 30 μg for microsome, 5 μg for the GFP detection, 1 μg for V-ATPase, H^+^-ATPase and Bip detection. Full images of western blot were shown in Supplementary Fig. 15b.

### Xylem sap and root cell sap analysis

Collection of xylem sap was performed as previously described with modifications^51^. WT and *oshma4* plants were hydroponically grown in a growth chamber for 5 weeks in half-strength Kimura B solution with Cu omitted. The plants were then transferred to a nutrient solution containing either 0.2 or 2 μM added CuSO_4_. After 1 week of treatment, the shoots were cut with a razor at about 2 cm above the root–shoot junction. The xylem sap was collected for 1 h after cutting. The first drop of xylem sap emerging was discarded to prevent contamination of the contents from damaged cells. Xylem sap from 16 plants was combined as one replicate and three replicates were made. The Cu concentration in the xylem sap was determined by ICP-MS. Root cell sap was prepared according to Ueno *et al*.^28^ with modifications. Briefly, WT and *oshma4* mutant plants were grown hydroponically in half-strength Kimura B nutrient solution for 2 weeks and transferred to nutrient solution containing either 0.2 or 2 μM CuSO_4_ for 7 days. Root cell sap was prepared from the root tips as follows. After washing entire root systems three times with 0.5 mM CaCl_2_, the first 1–2 cm of root tip segments were cut, rinsed with Milli-Q water and blotted dry with tissue paper. One root segment from each of 16 plants comprised a single sample, and a total of 4 samples were run per Cu growth treatment. Each sample of 16 root segments were put in a 0.22 μm filter unit (Ultrafreer-MC; Millipore) and centrifuged at 3,000*g* for 10 min at 4 °C to remove the apoplastic solution. Root segments were then frozen at −80 °C overnight. After thawing at room temperature for a short time, samples were centrifuged at 20,600*g* for 10 min to collect the root cell sap solution. Five microliter of each root cell sap sample were digested with 1 ml concentrated HNO_3_ at 118 °C for 1 h and the Cu concentration was determined using ICP-MS.

### Cu tolerance evaluation

To compare the Cu tolerance of WT and the *oshma4* mutant, seeds of each were soaked in tap water for 2 days at 30 °C and then transferred to a net floating on a 0.5 mM CaCl_2_ solution for 3 days. At day 4, seedlings were exposed to a 0.5 mM CaCl_2_ solution (pH 5.6) containing either no added CuSO_4_ or CuSO_4_ added at 100, 200, 400, 600 nM for 24 h. The experiment was performed at 25 °C. The root length of each seedling was measured before and after the treatments, and relative root elongation (=(root elongation with Cu)/(root elongation without Cu) × 100) was calculated. Twelve seedlings for each treatment were used. A long-term treatment with excess Cu was also performed by exposing WT, the *oshma4* mutant and two independent transgenic complementation lines to a nutrient solution with or without 2 μM added CuSO_4_ for 15 days at 25 °C. Shoot length was recorded and the normalized increased plant height was calculated as (plant height increase with Cu)/(plant height increase without Cu) × 100.

### Quantitative real-time PCR

To investigate the expression pattern of *OsHMA4* at different growth stages, different tissues from plants (cv Nipponbare) grown in a paddy field were harvested for RNA extraction and cDNA preparation^18^. The tissue-specific expression of *OsHMA4* in roots was examined with the help of laser microdissection according to the described methods^18^. The relative expression of *OsHMA4* was investigated by quantitative real-time RT-PCR using the *HistoneH3* gene as the internal control. To determine the expression of *OsHMA4* in response to different metals in the nutrient solution, total RNA was extracted from shoots and roots using a TRIzol Plus RNA Purification kit (Invitrogen, Life Technologies), and then treated with a PureLink DNase Set (Invitrogen, Life Technologies) to remove potential genomic DNA contamination. The cDNA synthesis was carried out using a SuperScript VILO cDNA Synthesis Kit (Invitrogen, Life Technologies). Quantitative real-time PCR was performed on an ABI StepOnePlus Real-Time PCR System (Applied Biosystems) with Maxima SYBR Green qPCR Master Mixes (Thermo Scientific). Ct values were normalized to the corresponding endogenous control gene (LOC_Os03g50885). The ΔΔCt method was used for quantitative RT-PCR analysis. The primer sequences are listed in Supplementary Table 3. Total RNA was extracted from yeast cells using a PureLink RNA Mini Kit (Thermo Fisher Scientific). The cDNA synthesis and quantitative real-time PCR was performed was performed as above. The yeast housekeeping gene *AGL9* was used as an endogenous control gene.

### Expressing *OsHMA4* in *Arabidopsis AtHMA5* mutant and WT Col-0

The construction of the *35S:GFP-OsHMA4(TQ)* and *35S:GFP-OsHMA4(LM)* vectors was described above. To generate the *AtHMA5* promoter driven *OsHMA4* expression vectors, the 2,900-bp promoter sequence of *AtHMA5* was PCR amplified from the genomic DNA of Col-0 and ligated into the *Pst*I-*Sal*I site of the pCambia1301 vector. The fragments containing *OsHMA4* coding sequence fused in-frame to GFP were released from the *35S:GFP-OsHMA4(TQ*) or *35S:GFP-OsHMA4(LM*) vectors and inserted into the *Sal*I-*Eco*RI site of above vector (*pCambia1301-AtHMA5pro)* to generate the *AtHMA5pro:GFP-OsHMA4(TQ)* and *AtHMA5pro:GFP-OsHMA4(LM)* vectors. The resulting plasmids were transformed into *A. tumeraciens* strain GV3101. The *35S:GFP-OsHMA4(TQ*) or *35S:GFP-OsHMA4(LM*) were introduced into the *Arabidopsis athma5* mutant (SALK_040252) or Col-0 and *AtHMA5pro:GFP-OsHMA4(TQ)* and *AtHMA5pro:GFP-OsHMA4(LM)* were introduced into the *athma5* mutant as described above. For the Cu tolerance assay, T3 transgenic plants were grown on MGRL medium containing 50 mg ml^−1^ hygromycin for 3 days and the positive plants were transferred to hygromycin-free MGRL medium containing 1 or 50 μM added CuSO_4_ for 7 days. To quantify tolerance to Cu in the growth medium root length was measured by marking the position of root tips on the Petri dish at the indicated times.

### Functional analysis of *OsHMA4* in yeast

The yeast (*Saccharomyces cerevisiae*) WT strain BY4741 (MATa his3Δ1 leu2Δ0 met15Δ0 ura3Δ0), BY4741-derived mutants *ccc2* (MATa his3Δ1 leu2Δ0 met15Δ0 ura3Δ0 YDR270w::kanMX4) and *ctr1* (MATa his3Δ1 leu2Δ0 met15Δ0 ura3Δ0 YPR124w::kanMX4) were purchased from Open Biosystems (http://dharmacon.gelifesciences.com/openbiosystems). To generate the yeast expression vector, the full-length coding sequence of *OsHMA4* was amplified from cDNA synthesized from TQ and LM and the CDS was ligated into the *Bam*HI–*Eco*RI site of pYES2 vector. To generate the pYEC2-OsHMA4(TQ)-GFP and pYEC2-OsHMA4(LM)-GFP vectors, the *OsHMA4* CDS was released from pYES2-OsHMA4(TQ) and pYES2-OsHMA4(LM) and ligate to the *Bam*HI–*Eco*RI site of pYEC2/CT–GFP^52^. To generate the pYES2-OsHMA4(TQ)-GFP and pYES2-OsHMA4(LM)-GFP vectors, the coding sequence of GFP was released from pYEC2/CT–GFP and ligated to the *Xba*I site of pYES2-OsHMA4(TQ) and pYES2-OsHMA4(LM). The direction of GFP was confirmed by sequencing. The expression of *OsHMA4* is under the control of a galactose-inducible promoter in these vectors. The resulting plasmids and empty vectors were transformed into various yeast strains using a Frozen-EZ Yeast Transformation II Kit (ZYMO Research). For complementation of the *ccc2* mutant, BY4741 and *ccc2* were transformed with pYES2 empty vector, pYES2-OsHMA4(TQ) or pYES2-OsHMA4(LM) and cultured at 30 °C overnight in 3 ml of SD-Ura media (6.7 g l^−1^ yeast nitrogen base, 1.92 g l^−1^ dropout mix without uracil) containing 2% (w/v) glucose. Cells were washed twice with 10 ml sterile deionized water and the optical density at 630 nm adjusted to 0.2 with sterile distilled water. After sequential 10-fold dilutions, 10 μl of cell suspensions of each genotype were spotted on Fe-limited, Fe-sufficient and Cu-sufficient media, respectively, and the plates incubated at 30 °C for 3 days. Fe-limited media was prepared as previously described^53^, and contained 0.17% (w/v) yeast nitrogen base without CuSO_4_ and FeCl_3_ (BIO 101 Systems), 0.2% (w/v) dropout mix without uracil, 2% (w/v) galactose, 1% (w/v) raffinose, 50 mM MES (pH 6.1), 1 mM 3-(2-pyridyl)-5, 6-bis (4-sulfophenyl)-1, 2, 4-triazine disodium salt (Ferrozine disodium salt; Sigma), 50 μM Fe(NH_4_)_2_(SO_4_)_2_, 1 μM CuSO_4_ and 2% (w/v) agar. The Fe-sufficient and Cu-sufficient media were modified from Fe-limited media by increasing the concentration of added Fe(NH_4_)_2_(SO_4_)_2_ and CuSO_4_ to 350 and 500 μM, respectively, and the Ferrozine disodium salt was omitted. For the metal tolerance assays, diluted cell suspensions were prepared as above and spotted on the SD-Ura media containing 2% (w/v) galactose, 1% (w/v) raffinose and indicated metals. The growth of the BY4741 strain transformed with various plasmids in liquid SD-Ura media containing Cu was determined on 2 ml 96-well deep plates. Overnight yeast cells were prepared as above and the optical density at 630 nm was adjusted to 0.5 with sterile distilled water. Fifty microliter of cell suspensions was added to 1 ml SD-Ura media in each well containing 2% (w/v) galactose, 1% (w/v) raffinose and 0, 2.5 or 3 mM CuSO_4_. The plates were incubated at 30 °C and shaken at 400 r.p.m. The optical density at 630 nm was determined at indicated time using a plate reader.

### Homology modelling and sequence alignment

Homology modelling was conducted using the web-based SWISS-MODEL platform (http://swissmodel.expasy.org)^54^. The crystal structure of a *Legionella pneumophila* P-type ATPase CopA (PDB ID: 3RFU)^22^ was used as a template. The models of OsHMA4 from TeQing and Lemont were built separately using the same default parameters. The model quality was evaluated using the structure assessment tools of the Swiss-Model workspace, including ANOLEA^55^, DFire^56^ and QMEAN^57^. Structures were viewed using the DeepView/Swiss-PdbViewer 4.1 (http://www.expasy.org/spdbv/)^58^. Multiple sequence alignments of HMA proteins were conducted in BioEdit software using the ClustalW method.

### Genotyping of USDA rice core collection

The seeds of USDA Rice Core Collection were obtained from the Genetic Stocks Oryza (GSOR) Collection, USDA-ARS. In total, 1,349 accessions of the USDA Rice Core Collection were germinated and DNA was extracted for genotyping. Nine dCAPS markers were developed based on the non-synonymous polymorphisms in the coding sequence of *OsHMA4* identified from 950 different rice genomes^29^. The primer sequences are listed in Supplementary Table 3. Five of these polymorphisms were confirmed to occur in the USDA Rice Core Collection. Among these five polymorphic sites, four of them had a minor allele frequency greater than 0.05, which was further used to evaluate the contribution of *OsHMA4* in controlling grain Cu in the population. A linear model was used to assess the proportion of variation in grain Cu in plants grown in flooded and unflooded conditions explained by the four polymorphic sites with a minor allele frequency >0.05. The linear model included each polymorphic site as explanatory variables. To account for potential non-independence resulting from cryptic kinship between accessions, relative kinship among accessions was calculated using by SPAGeDi software^59^ based on 84 SSR markers^60^, and the Loiselle coefficient^61^ was used to create the pair-wise kinship matrix. All negative values were set to zero^62^. A principal components analysis of the kinship matrix was performed and the eigenvectors of the first four principal components were included as explanatory variables in the linear model^63^. The first four principal components explained 87% of the variation in the kinship matrix. Only the accessions with the genotype available at all four polymorphic sites, genotyped by 84 SSR markers, and grain Cu concentration were included in the analysis, resulting in a total of 1,241 accession under flooded condition and 1,210 accessions in unflooded condition.

The map image used in Fig. 6 was generated using R package rworldmap^64^ with data derived from Natural Earth v1.4.0 (http://www.naturalearthdata.com/).

### Data availability

The authors declare that all data supporting the findings of this study are available within the article and its Supplementary Information files or are available on request from the corresponding authors.

## Additional information

**Accession codes**: The sequences of *OsHMA4* from TeQing and Lemont have been deposited in GenBank nucleotide database under the accession code KU168831 and KU168832, respectively.

**How to cite this article:** Huang, X.-Y. *et al*. A heavy metal P-type ATPase OsHMA4 prevents copper accumulation in rice grain. *Nat. Commun.* 7:12138 doi: 10.1038/ncomms12138 (2016).

## Supplementary Material

Supplementary Information Supplementary Figures 1 - 15 and Supplementary Tables 1 - 3
