# Supplementary material for: A heavy metal P-type ATPase OsHMA4 prevents copper accumulation in rice grain
Source: Nat Commun. 2016 Jul 8;7:12138. doi: 10.1038/ncomms12138 (PMC4941113; doi:10.1038/ncomms12138)
Supplement: Supplementary Information — Supplementary Figures 1 - 15 and Supplementary Tables 1 - 3 [file ncomms12138-s1.pdf]

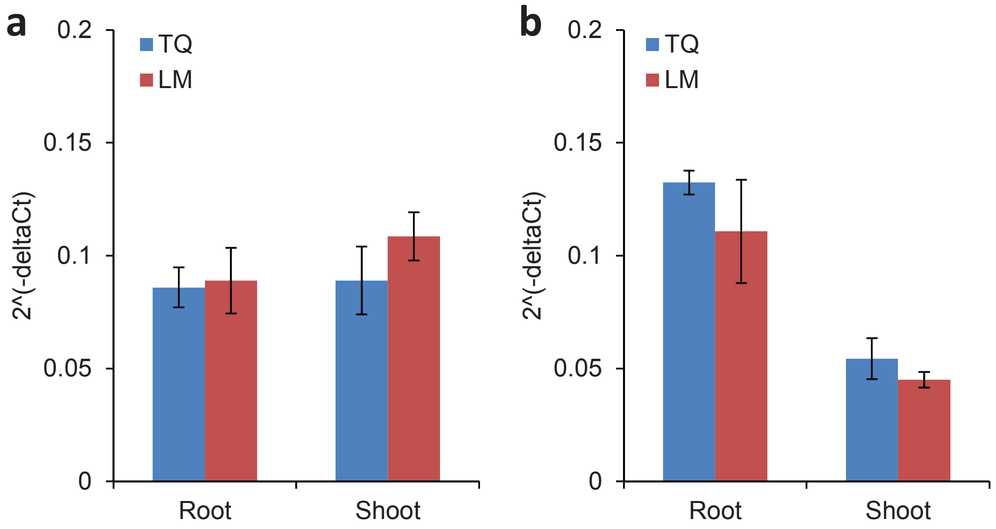

Supplementary Figure 1. Expression of *OsZTP29* and *OsHMA4* in TeQing (TQ) and Lemont (LM). Plants were grown hydroponically in half strength Kimura B nutrient solution for two weeks. Roots and shoots were harvested separately for RNA extraction. Expression level of *OsZTP29* (**a**) and *OsHMA4* (**b**) was quantified by qRT-PCR and normalized to the rice actin gene. Data are presented as mean ± SD (*n* = 3).

Supplementary Figure 2

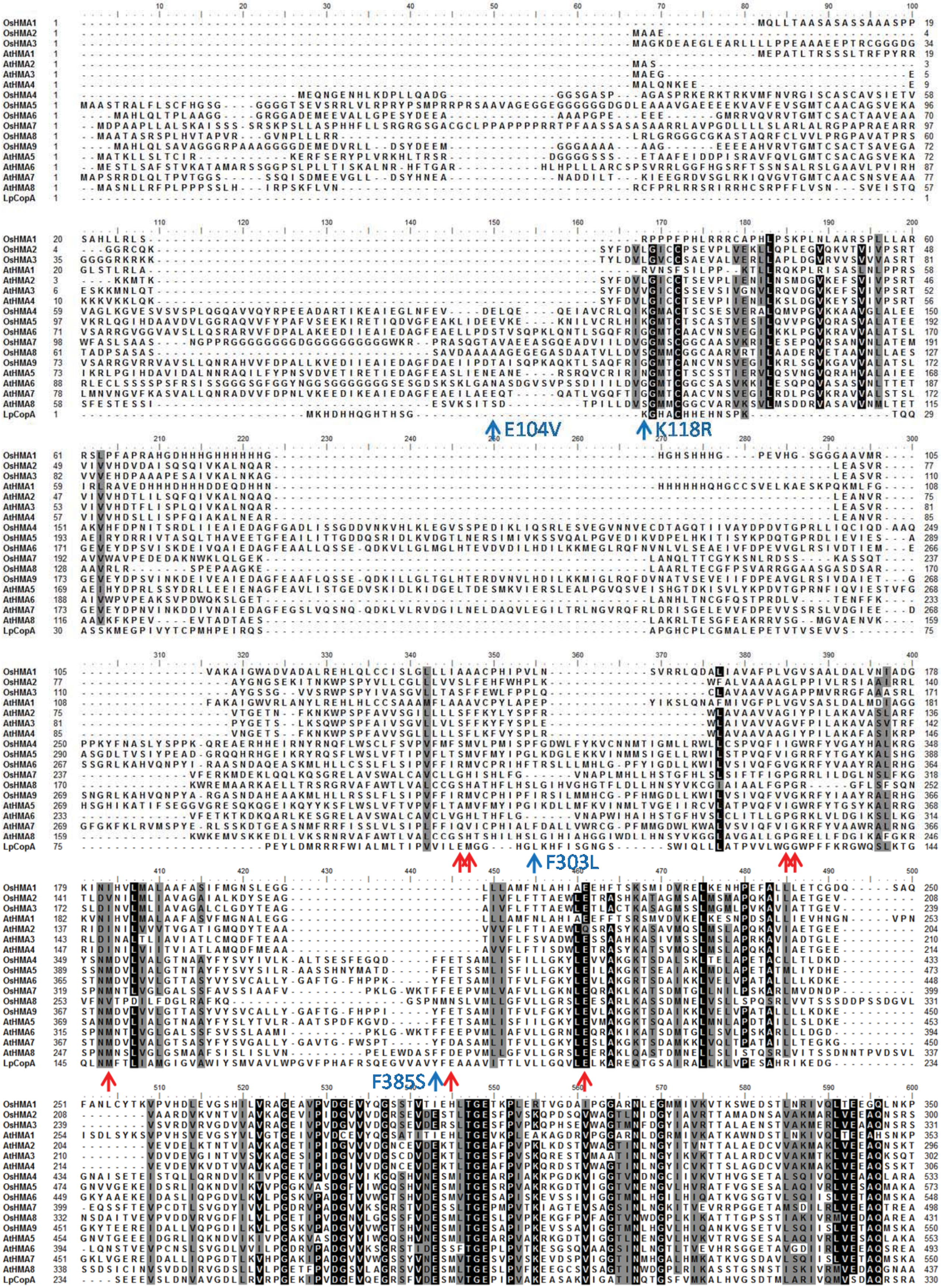

Supplementary Figure 2 (continued)

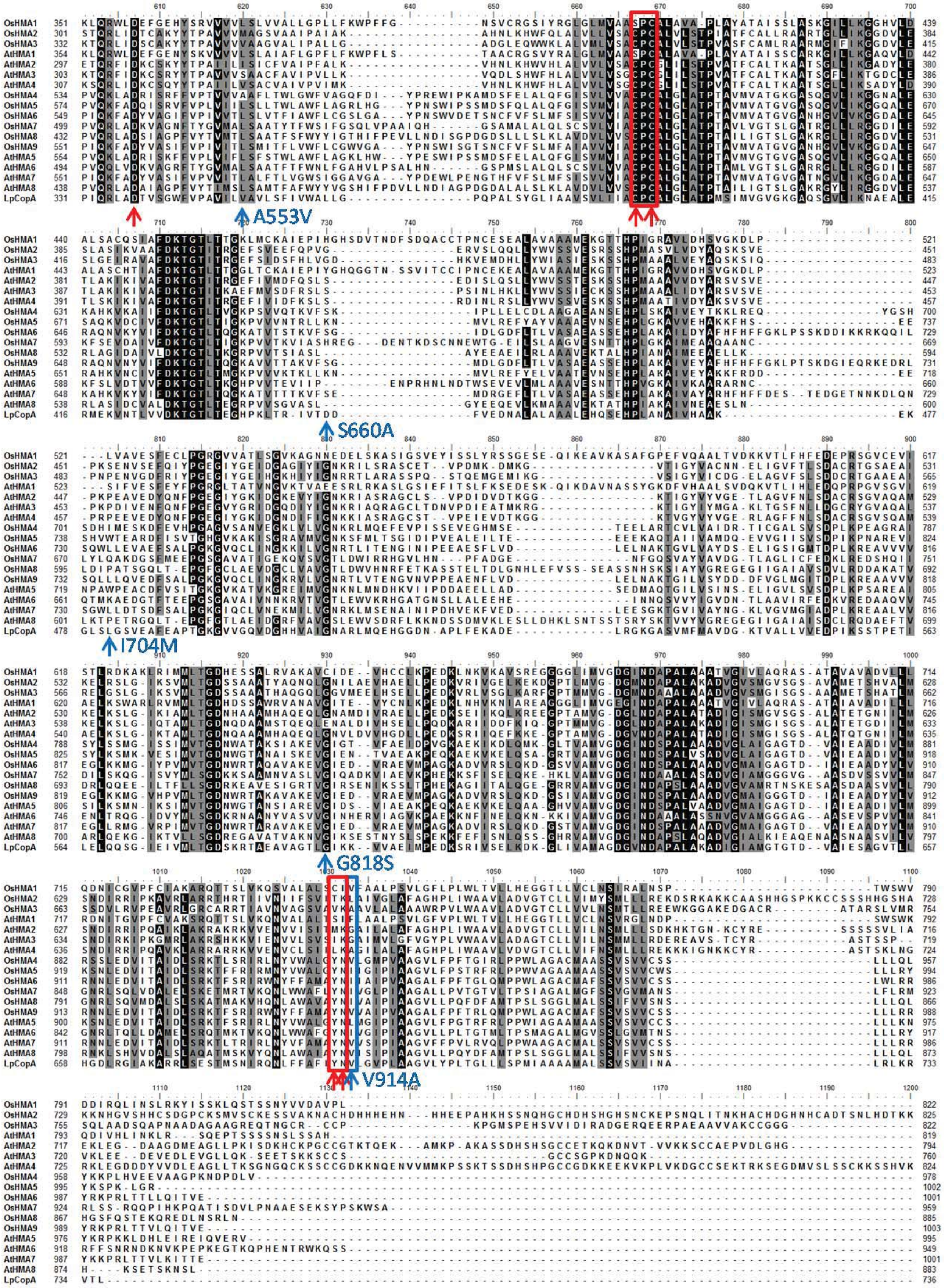

Supplementary Figure 2(continued)

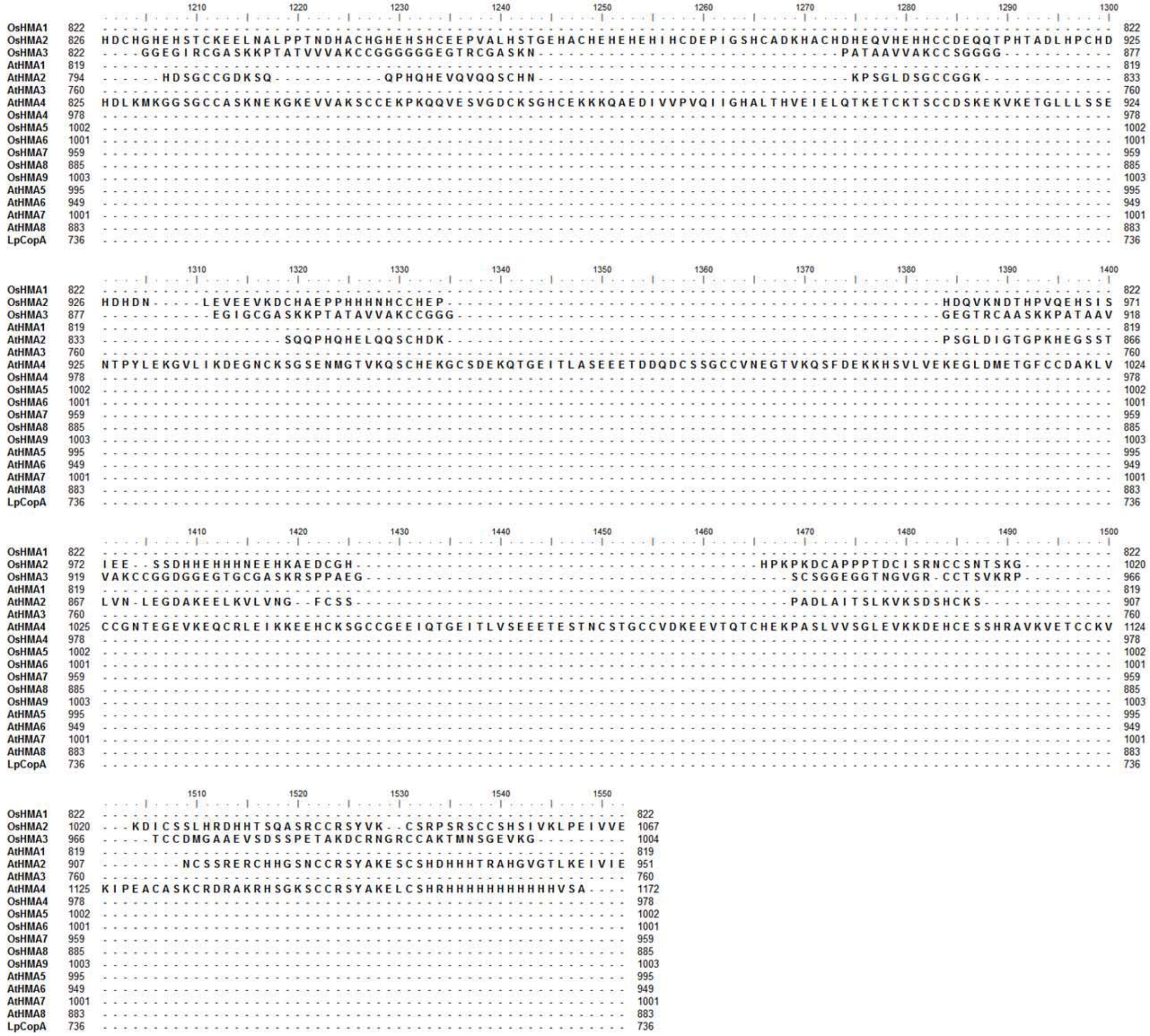

Supplementary Figure 2. Amino acid sequence alignment of *Legionella pneumophila* P_{IB}-type copper transporting ATPase LpCopA and heavy metal P_{IB}-type ATPases from *Arabidopsis thaliana* and rice. Sequence alignment was performed using Clustal W. Identical and similar residues are displayed in black or grey background. The membranous binding sites of copper based on the crystal structure of LpCopA are marked with red arrows. The polymorphic amino acids of OsHMA4 among USDA core collection are marked with blue arrows. The polymorphic V914A between TeQing and Lemont is highlighted with a blue box. The conserved YN and CPC motif are highlighted with red boxes.

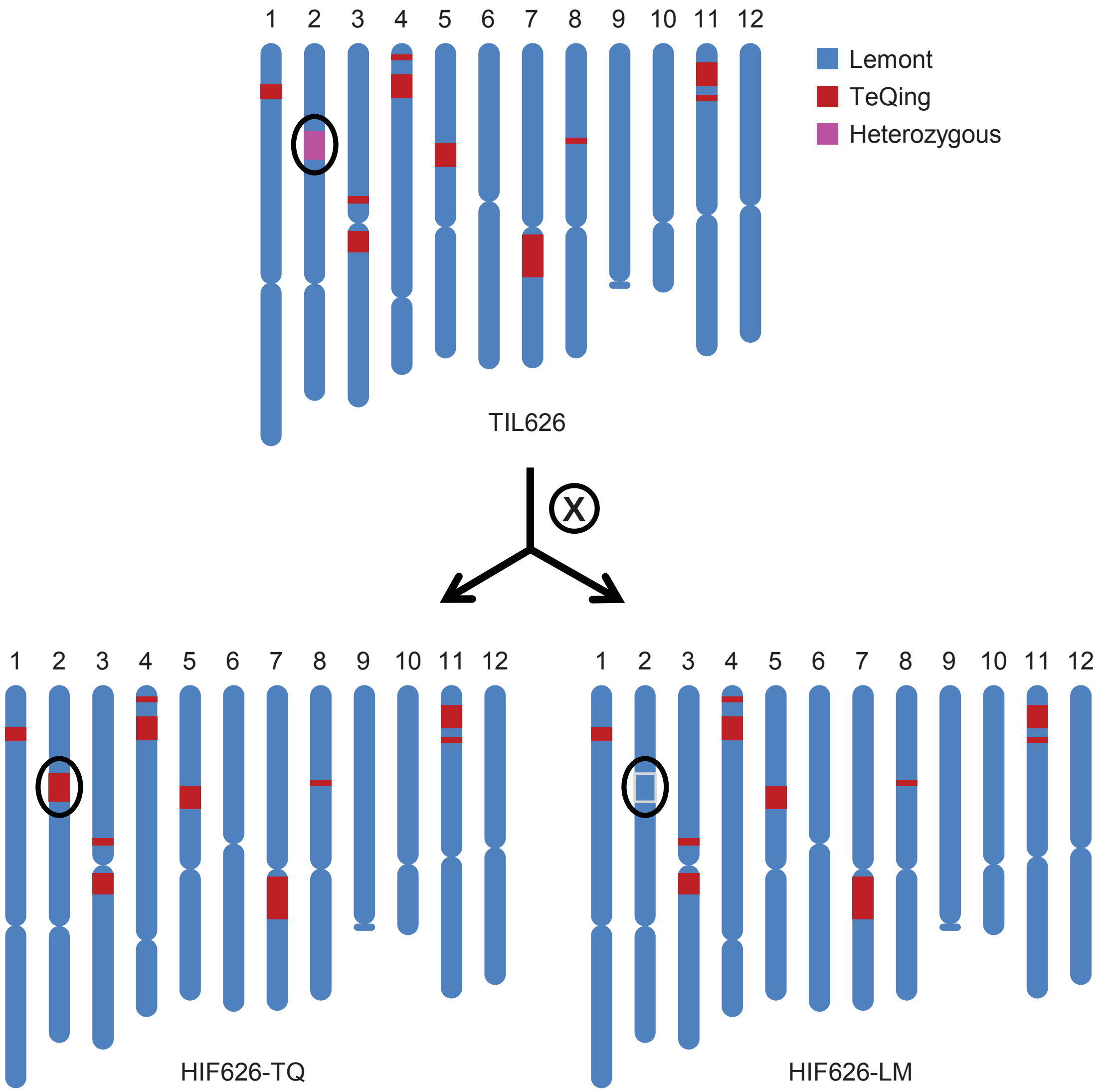

Supplementary Figure 3. Schematic diagram of development of heterogeneous inbred families (HIF). A dCAPS marker was developed based on the single nucleotide polymorphism (SNP) site that causes V914A variation between TQ and LM, and was used for genotyping the TILs at the BC_4F_9 generation. The line TIL626 was determined to be heterozygous at the *OsHMA4* locus. TIL626 was self-pollinated and two lines were isolated in the next generation which are homozygous with *OsHMA4* alleles from TQ or LM, respectively, namely HIF626-TQ and HIF626-LM. The black oval highlights the genomic region where *OsHMA4* localizes. Columns in blue, red and magenta represent the TQ, LM or heterozygous genomic fragments.

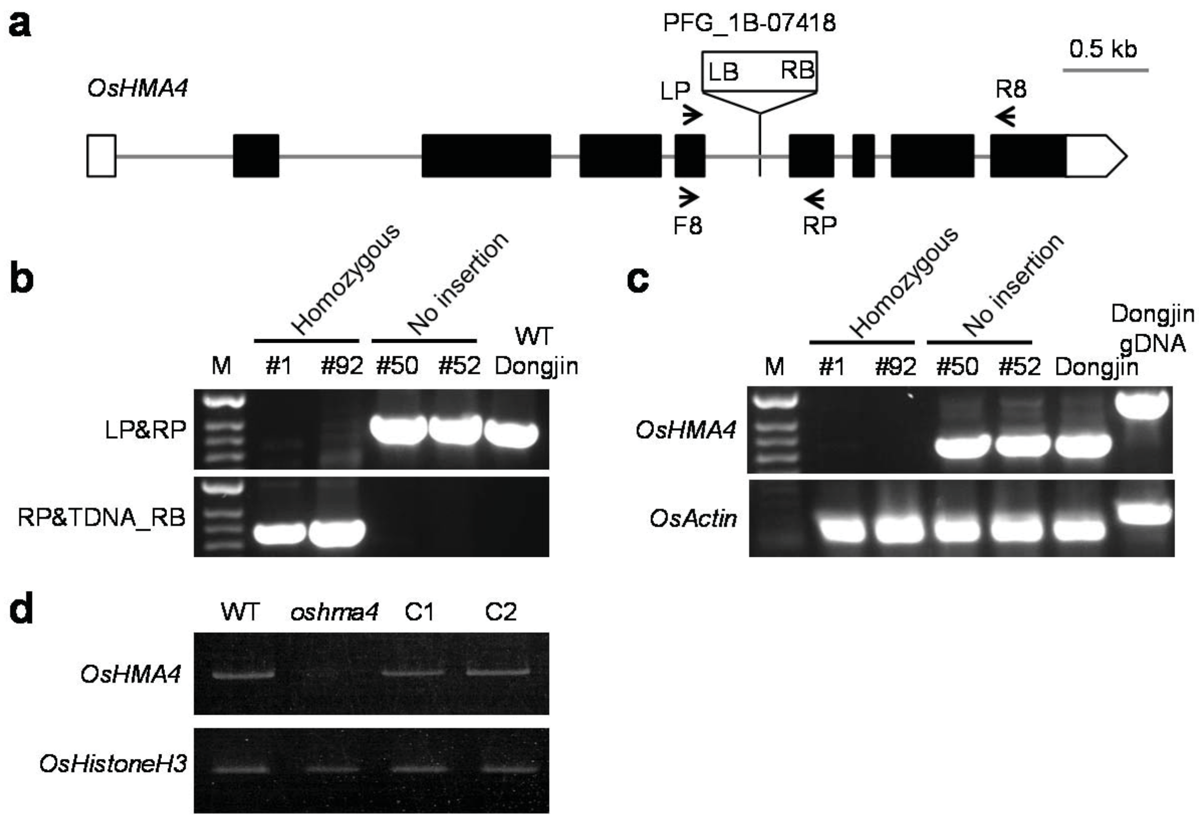

Supplementary Figure 4. Molecular characterization of T-DNA insertion mutant of *OsHMA4* and the transgenic complementation lines. (**a**) Gene structure of *OsHMA4*. Black bars, grey lines and white bars represent exons, introns and untranslated regions, respectively. T-DNA insertion sites are indicated by the rectangle. The primers using for genotyping and RT-PCR are shown as arrows. (**b**) Genotyping T-DNA insertion lines. Gene specific primers (FP and RP) and right border primer of the T-DNA insertion (RB) were used. Two plants of homozygous lines and non T-DNA insertion lines were genotyped. The background parent line Dongjin was used as control. (**c**) Determination of the expression of *OsHMA4* in the T-DNA lines by RT-PCR. The cDNA and genomic DNA of Dongjin were used as controls. *OsActin* gene was used as the internal control. (**d**) Expression of *OsHMA4* in WT, *oshma4* and two independent transgenic complementation lines (C1, C2). RNA was isolated from the whole plants and expression level of *OsHMA4* we determined by RT-PCR. *OsHistoneH3* was used as loading control.

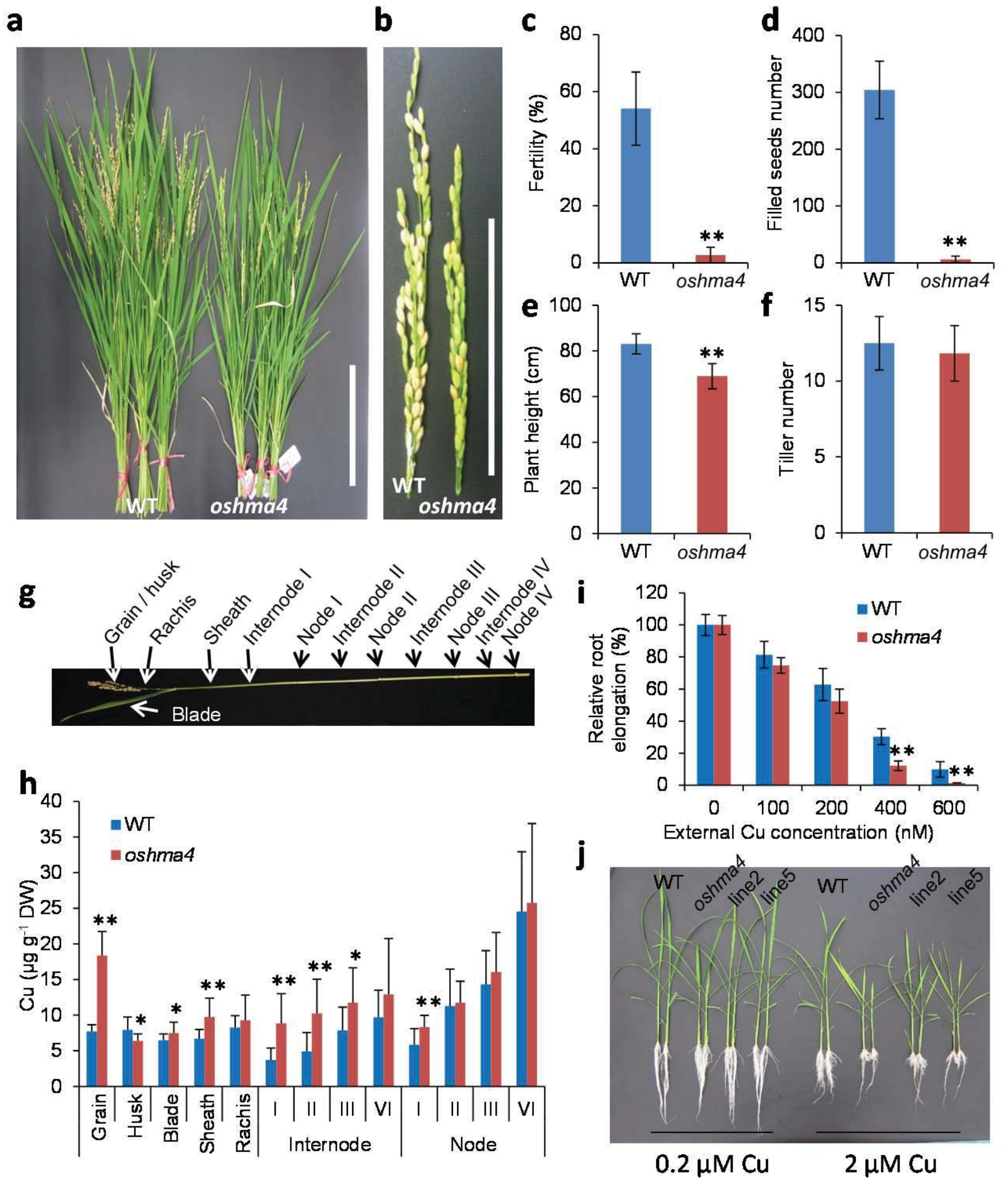

Supplementary Figure 5. Characterization of *oshma4* mutant and complementation of *oshma4* mutant . (**a**) Growth of wild-type (WT) and *oshma4* in the field at harvesting stage. Bar, 20 cm. (**b**) Panicles of WT and *oshma4* mutant. Bar, 10 cm. (**c**) Fertility of WT and *oshma4*. (**d**) The number of filled grain per plant. Filled seeds were chosen by soaking the seeds in 8.5% (w/v) NaCl solution. (**e**) Plant height. (**f**) Number of tillers. (**g**) Different tissues of the main tiller sampled for analysis at harvesting stage. (**h**) Cu concentration in different tissues of WT and *oshma4*. (**i**) Knockout of *OsHMA4* resulted in hypersensitivity to excess Cu. Seedlings of WT and *oshma4* were exposed to the solutions containing different concentrations of Cu (0, 100, 200, 400 and 600 nM) for 24 hours. The root length was measured before and after treatment. Data are presented as means ± SD (n = 6 in (**c** – **f**) and n = 12 in (**h**) and (**i**)). * and ** indicate significant difference between WT and *oshma4* mutant at $P \leq 0.05$ and $P \leq 0.01$, respectively (Student's t test). DW, dry weight. (**j**) The image of WT, *oshma4* and two independent transgenic lines expressing of *GFP-OsHMA4* under control of *OsHMA4* native promoter in the *oshm4* mutant. Plants were grown in nutrient solution with 0.2 or 2 µM $CuSO_4$ for 10 days..

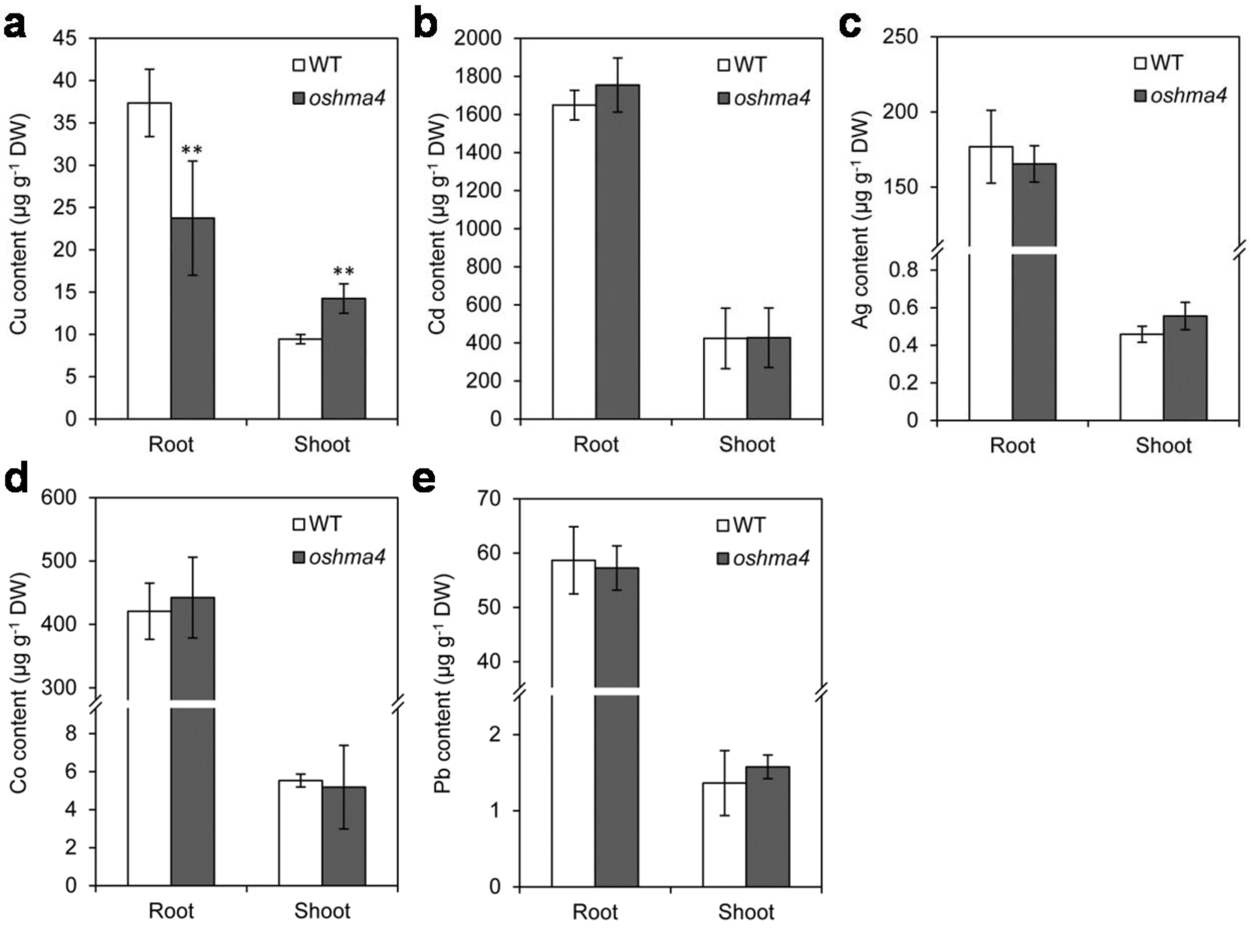

Supplementary Figure 6. OsHMA4 is not involved in the compartmentalization of Cd, Ag, Co and Pb. WT and *oshma4* plants were hydroponically grown in half strength Kimura B nutrient for fifteen days and treated without (**a**) or with 0.2 μM $Cd(NO_3)_2$ (**b**), 0.2 μM $AgNO_3$ (**c**), 5 μM $CoCl_2$ (**d**) or 0.2 μM $Pb(NO_3)_2$ (**e**) for one week. The Cu (**a**) in the roots and shoots of control plants and Cd (**b**), Ag (**c**), Co (**d**) and Pb (**e**) in the roots and shoots of treated plants were quantified by ICP-MS. Data are presented as means ± SD (n = 4). ** indicates significant difference between WT and the *oshma4* mutant at $P \leq 0.01$ (Student's t test). DW, dry weight.

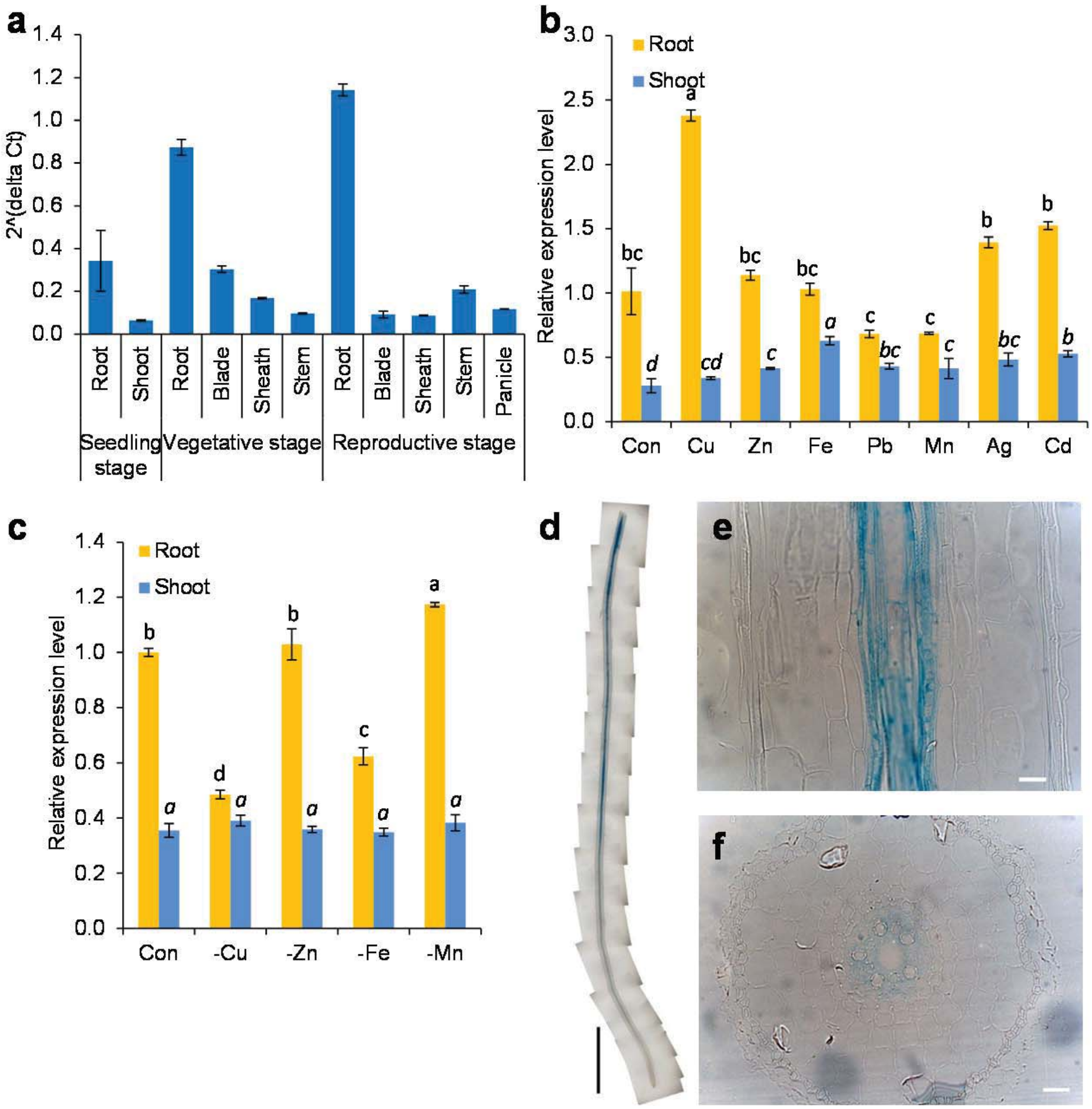

Supplementary Figure 7. Expression patterns of *OsHMA4* in the roots and expression of *OsHMA4* under different heavy metals treatments and element deficiency treatments. (**a**) Expression level of *OsHMA4* in different organs at different growth stages of TeQing. Plants were grown in a greenhouse. (**b**) Expression of *OsHMA4* under different heavy metals treatments. Two-week-old TQ plants were treated with 20 μM Cu, 20 μM Zn, 2 mM Fe, 20 μM Pb, 20 μM Mn, 20 μM Ag or 20 μM Cd for 5 days, respectively. (**c**) Expression of *OsHMA4* under element deficiency treatment. TQ plants were grown in half strength Kimura B nutrient solution for one week and treated with Cu, Zn, Fe or Mn free nutrient solution for 7 days, respectively. Expression level of *OsHMA4* was determined by qRT-PCR in roots and shoots separately. Data are presented as mean ± SD (n = 3). Con, control. Columns with different italic or non-italic letters in (**b**) and (**c**) indicate significant difference in shoots and roots, respectively ($P \leq 0.01$, Fisher's LSD test). (**d** - **f**) *OsHMA4* promoter-GUS expression patterns of *OsHMA4* in the roots of transgenic rice plants. GUS signal was observed in the vascular system of roots. Plants were grown in nutrient solution containing 0.2 μM $CuSO_4$. Bars are 0.5 cm in (**d**), 20 μm in (**e**) and (**f**).

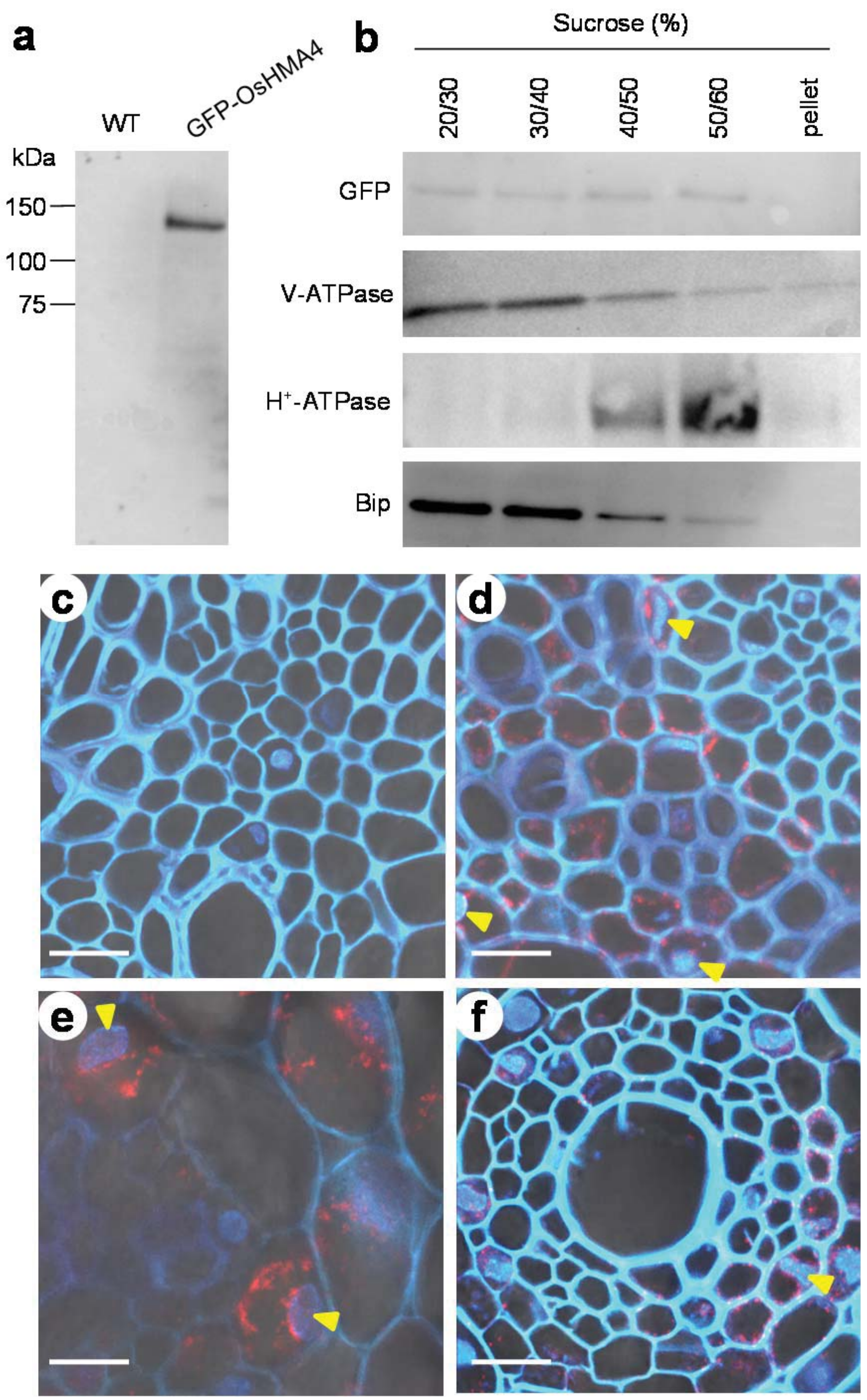

Supplementary Figure 8. Localization of OsHMA4 in roots of transgenic rice plants expressed *GFP-OsHMA4* driven by the native promoter of *OsHMA4* . (**a, b**) Western blot analysis using anti-GFP antibody. Specificity of anti-GFP antibody was determined in the transgenic plants (**a**). WT (Dongjin) was used as negative control. (**b**) Sucrose-density gradient analysis. Microsome was extracted from whole roots of WT or transgenic plants. The microsome fraction was fractionated by sucrose-density gradient. Western blot was performed using polyclonal antibodies of anti-GFP, anti-V-ATPase (tonoplast marker), anti-H^+-ATPase (plasma membrane marker), or anti-Bip (ER marker). (**c–f**) Immunostaining of GFP-OsHMA4 with anti-GFP antibody in WT (**c**) and transgenic plants (**d–f**). (**c**) Cross section of the basal node of WT; (**d–e**), cross section of the basal node of transgenic plants; (**f**) cross section of roots of transgenic plants. Plants were cultured without Cu for 2 weeks and then treated with 0.2 (**c–e**) and 2 μM (**f**) Cu for 24 h. The signal of anti-GFP was shown red. Blue color indicates auto-fluorescence of cell wall and nuclei (yellow arrows) were stained by DAPI. Bars in (**c–f**), 10 μm.

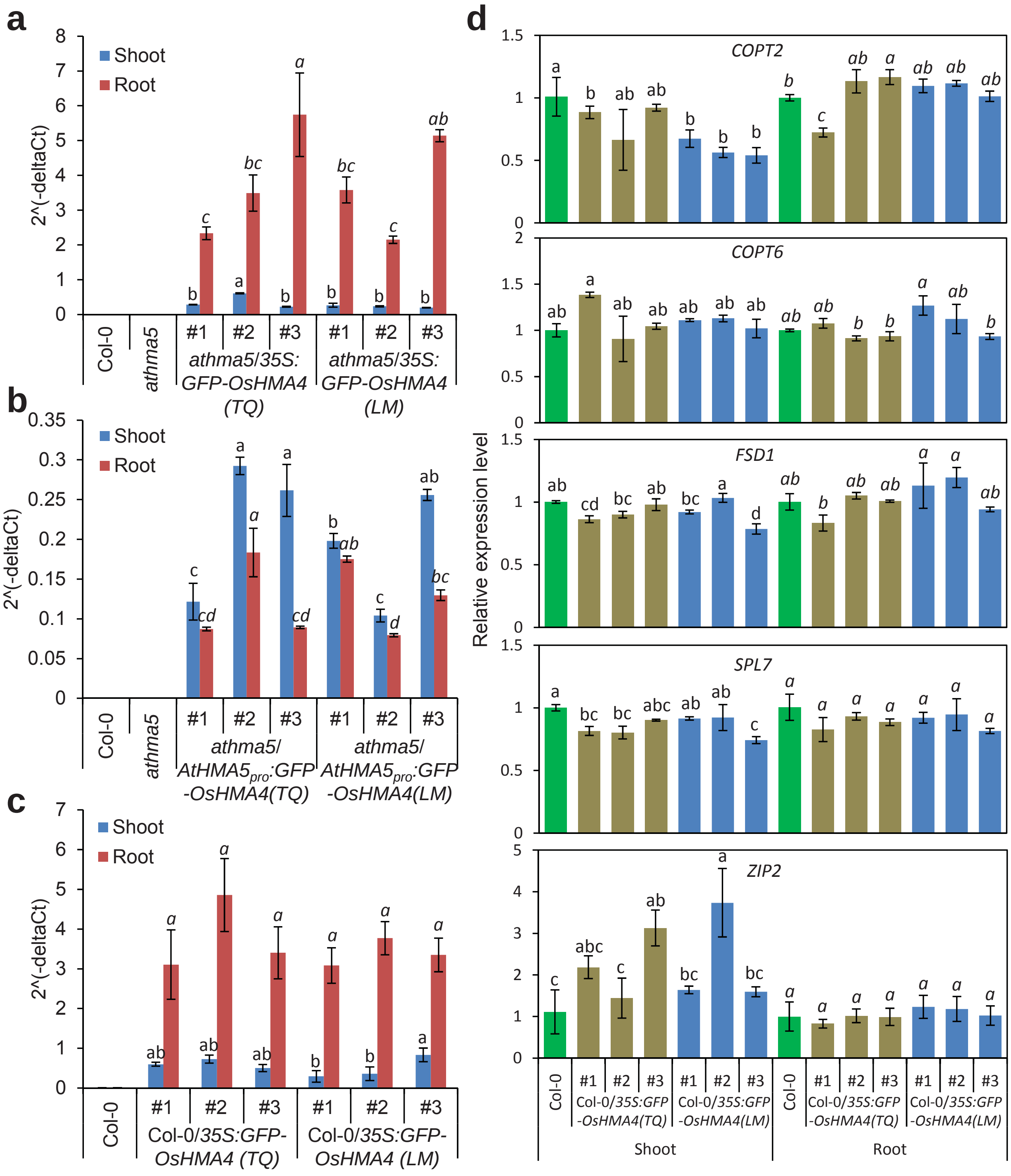

a
Shoot
Root
2^(-deltaCt)
0
1
2
3
4
5
6
7
8
b
c
a
bc
b
a
b
bc
c
b
ab
b
Col-0
athma5
#1
#2
#3
#1
#2
#3
athma5/35S:
GFP-OsHMA4
(TQ)
athma5/35S:
GFP-OsHMA4
(LM)
b
Shoot
Root
2^(-deltaCt)
0
0.05
0.1
0.15
0.2
0.25
0.3
0.35
c
cd
a
a
a
cd
b
ab
c
d
ab
bc
Col-0
athma5
#1
#2
#3
#1
#2
#3
athma5/
AtHMA5pro:GFP
-OsHMA4(TQ)
athma5/
AtHMA5pro:GFP
-OsHMA4(LM)
c
Shoot
Root
2^(-deltaCt)
0
1
2
3
4
5
6
7
ab
a
ab
a
ab
a
b
a
b
a
a
a
Col-0
#1
#2
#3
#1
#2
#3
Col-0/35S:GFP-
OsHMA4 (TQ)
Col-0/35S:GFP-
OsHMA4 (LM)
d
Relative expression level
COPT2
0
0.5
1
1.5
a
b
ab
ab
b
b
b
b
c
ab
a
ab
ab
ab
COPT6
0
0.5
1
1.5
2
ab
a
ab
ab
ab
ab
ab
ab
ab
b
b
a
ab
b
FSD1
0
0.5
1
1.5
ab
cd
bc
ab
bc
a
d
ab
b
ab
ab
a
a
ab
SPL7
0
0.5
1
1.5
a
bc
bc
abc
ab
ab
c
a
a
a
a
a
a
a
ZIP2
0
1
2
3
4
5
c
abc
c
ab
bc
a
bc
a
a
a
a
a
a
a
Col-0
#1
#2
#3
#1
#2
#3
Col-0/35S:GFP
-OsHMA4(TQ)
Col-0/35S:GFP
-OsHMA4(LM)
Col-0
#1
#2
#3
#1
#2
#3
Col-0/35S:GFP
-OsHMA4(TQ)
Col-0/35S:GFP
-OsHMA4(LM)
Shoot
Root

Supplementary Figure 9. Expression of *OsHMA4* and Cu regulated genes in *Arabidopsis* transgenic lines. (**a**) Expression of *OsHMA4* in *athma5* transformed with *OsHMA4* from TeQing (TQ) or Lemont (LM) driven by a CaMV 35S promoter. The same transgenic lines used in Fig. 4**b** were shown. Plants were grown on MGRL media containing 1 μM $CuSO_4$ for two weeks. (**b**) Expression of *OsHMA4* in *athma5* transformed with *OsHMA4* from TeQing (TQ) or Lemont (LM) driven by the *AtHMA5* native promoter. The same transgenic lines used in Fig. 4**c**, **d** were shown. Plants were grown on MGRL media with 1 μM $CuSO_4$ for 6 days and then transferred to the media containing 50 μM $CuSO_4$ for 10 days. (**c**) Expression of *OsHMA4* in Col-0 transformed with *OsHMA4* from TeQing (TQ) or Lemont (LM) driven by a CaMV 35S promoter. The same transgenic lines used in Supplementary Figure 11 were shown. Plants were grown on MGRL media containing 1 μM $CuSO_4$ for two weeks. (**d**) The relative expression level of Cu regulated genes *COPT2*, *COPT6*, *FSD1*, *SPL7*, and *ZIP2* in the transgenic lines expressing *OsHMA4* from TeQing (TQ) or Lemont (LM) by a CaMV 35S promoter in Col-0 background. Plants were grown on MGRL media containing 1 μM $CuSO_4$ for two weeks. Gene expression levels were determined by qRT-PCR. The *UBQ10* gene was used as the endogenous control. The relative expression levels of Cu regulated genes in the shoot and root in (**d**) were normalized to the shoot and root of Col-0, respectively. Data were shown as means ± SD (n = 3). Columns with different italic or non-italic letters in (**a**) to (**d**) indicate significant difference in shoots and roots, respectively ($P \leq 0.01$, Fisher's LSD test).

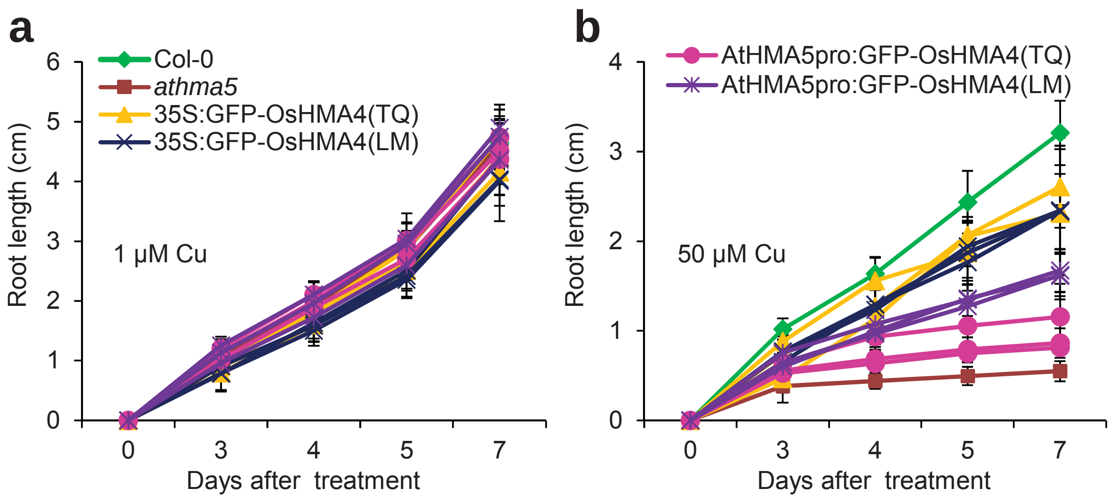

Supplementary Figure 10. Root length of *OsHMA4* transgenic lines grown on agar media with low or high Cu. Plants were grown on MGRL media with 1 µM Cu for 3 days and then transferred to media containing 1 µM (**a**) or 50 µM (**b**) $CuSO_4$. Root length was measured at 3, 4, 5 and 7 days after transfer. Three independent lines for each construct are shown. Data are shown as means ± SD (*n* = 9 to 16).

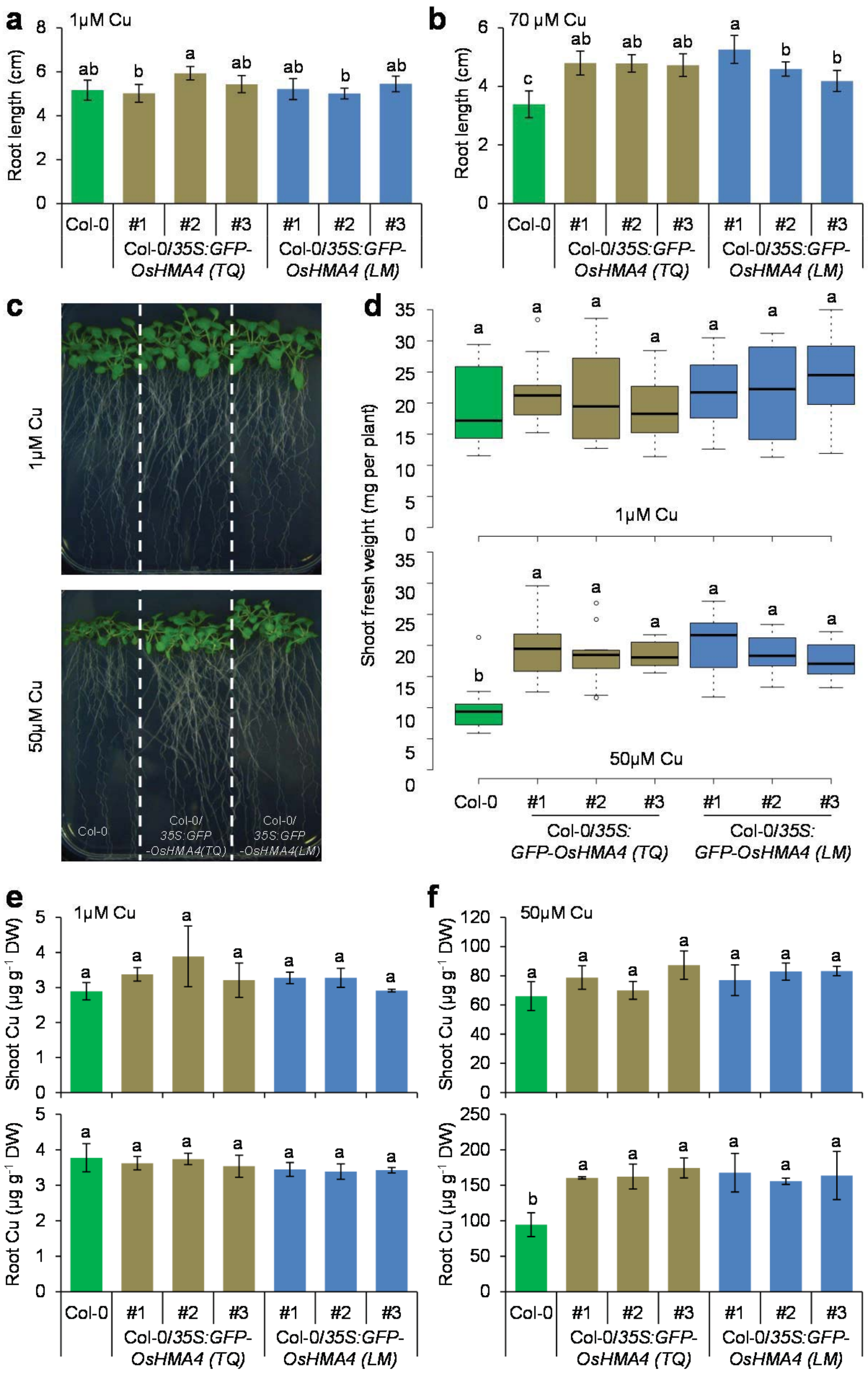
a
1μM Cu
Root length (cm)
ab
b
a
ab
ab
b
ab
Col-0
#1
#2
#3
Col-0/35S:GFP-OsHMA4 (TQ)
Col-0/35S:GFP-OsHMA4 (LM)
b
70 μM Cu
c
ab
ab
ab
a
b
b
c
1μM Cu
50μM Cu
Col-0
Col-0/35S:GFP-OsHMA4(TQ)
Col-0/35S:GFP-OsHMA4(LM)
d
Shoot fresh weight (mg per plant)
1μM Cu
50μM Cu
Col-0/35S:GFP-OsHMA4 (TQ)
Col-0/35S:GFP-OsHMA4 (LM)
e
1μM Cu
Shoot Cu (μg g-1 DW)
Root Cu (μg g-1 DW)
f
50μM Cu

44 **Supplementary Figure 11. Overexpression of *OsHMA4* in *Arabidopsis* Col-0 enhances tolerance to Cu stress.** (**a, b**) Root length of Col-0 transformed with *GFP-OsHMA4* from TeQing (TQ) or Lemont (LM) driven by CaMV 35S promoter. Plants were grown on MGRL media with 1 μM $CuSO_4$ for 4 days and then transferred to the media containing 1 (**a**) or 70 μM (**b**) $CuSO_4$. The root length was measured 8 days after transfer. Three independent lines for each construct were shown. Data are shown as means ± SD (n = 9). (**c**) Phenotype of *OsHMA4* overexpression lines in Col-0. Plants were grown on MGRL media with 1 μM $CuSO_4$ for 7 days and then transferred to the media containing 1 or 50 μM $CuSO_4$ for 10 days. (**d**) Shoot fresh weight of the plants in (**c**). Data are shown as boxplots (n = 12). (**e, f**) Cu concentration in shoots and roots of *OsHMA4* overexpression lines in Col-0. Plants were grown on the media containing 1 μM (**e**) or 50 μM $CuSO_4$ (**f**) as described in (**c**). Data are shown as means ± SD (n = 3 with 4 plants in each replicate). DW, dry weight. Columns or boxes with different letters in (**a**), (**b**), and (**d**) to (**f**) indicate significant difference ($P \leq 0.01$, Fisher's LSD test).

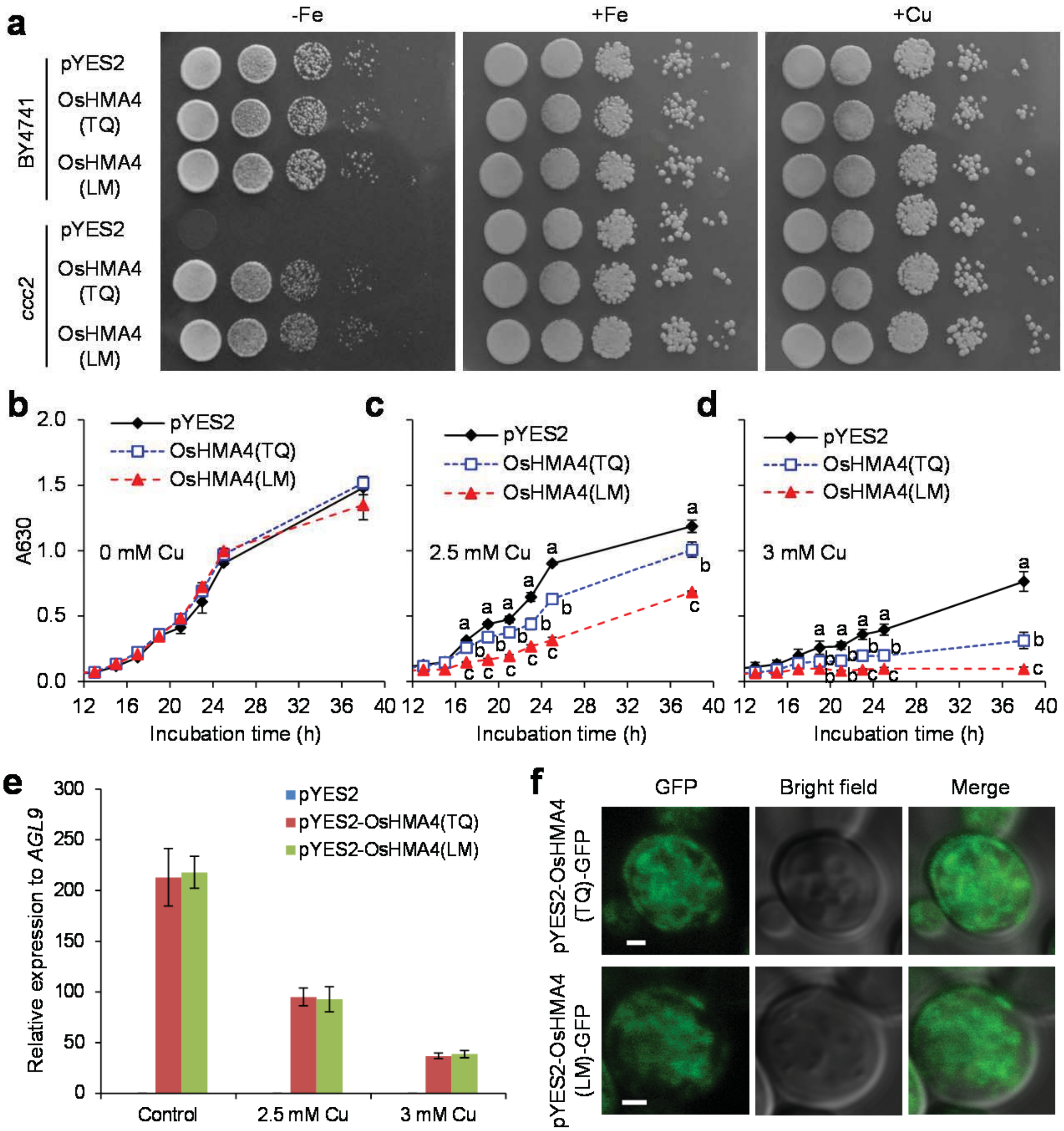
a
-Fe
+Fe
+Cu
BY4741
ccc2
pYES2
OsHMA4 (TQ)
OsHMA4 (LM)
b
c
d
pYES2
OsHMA4(TQ)
OsHMA4(LM)
A630
0 mM Cu
2.5 mM Cu
3 mM Cu
Incubation time (h)
e
Relative expression to AGL9
pYES2
pYES2-OsHMA4(TQ)
pYES2-OsHMA4(LM)
Control
2.5 mM Cu
3 mM Cu
f
GFP
Bright field
Merge
pYES2-OsHMA4 (TQ)-GFP
pYES2-OsHMA4 (LM)-GFP

Supplementary Figure 12. Expression and subcellular localization of OsHMA4 in yeast and the enhancement of yeast Cu tolerance by expressing OsHMA4 in a centromeric plasmid. (**a**) Cu transport activity of OsHMA4 in yeast. Wild-type yeast strain BY4741 and *ccc*2 mutant were transformed with empty vector pYES2 or *OsHMA4* from TeQing (*OsHMA4(TQ)*) or from Lemont (*OsHMA4(LM)*). Overnight cell suspension with serial dilutions (1:10) were spotted on Fe-limited (-Fe), Fe-sufficient (+Fe), and Cu-sufficient (+Cu) media and incubated at 30˚C for 3 days. (**b** - **d**) Expression of *OsHMA4* in yeast induced sensitivity to Cu stress. Growth curve of wild-type yeast strain BY4741 transformed with empty vector pYES2 or *OsHMA4* from TQ or LM. Absorbance at 630 nm (A630) of cultures containing without extra Cu added (**b**) or with 2.5 (**c**) or 3 mM (**d**) $CuSO_4$ added was measured at different time points as shown. Data were shown as means ± SD (n = 3). Different letters at each time points indicate significant difference between strains at this time point ($P \leq 0.01$, Fisher's LSD test). Time points without letters indicate no significant difference. (**e**) Expression of *OsHMA4* in the wild-type yeast strain BY4741 transformed with empty vector pYES2 or *OsHMA4* from TQ or LM. Yeast strains were grown in liquid media without (Control) or with 2.5 or 3 mM $CuSO_4$. The expression level was determined by qRT-PCR and normalized to a yeast housekeeping gene *AGL9*. Data were shown as means ± SD with three independent colonies. (**f**) Subcellular localization of OsHMA4 in yeast when expressing in a high-copy-number 2μ origin plasmid pYES2. GFP signals were observed throughout the cell. Bar, 1 μm.

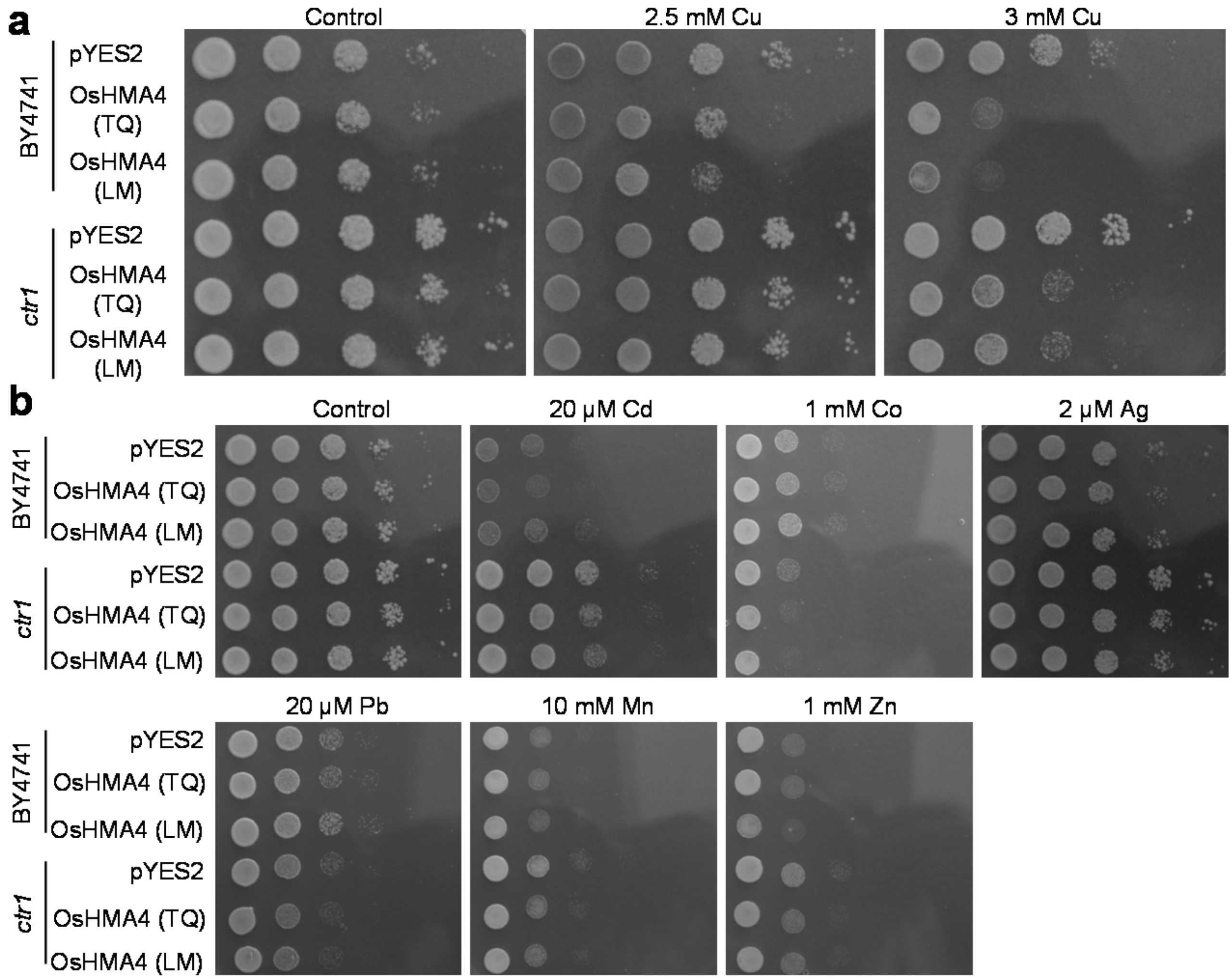

Supplementary Figure 13. Growth of yeast strains harbouring OsHMA4 on the media containing different heavy metals. (**a**) Expression of *OsHMA4* in yeast induced sensitivity to Cu stress. Overnight yeast cell suspension of BY4741 and *ctr*1 mutant transformed with empty vector pYES2 or *OsHMA4* from TQ or LM were serially diluted and spotted on the media without extra Cu added (control) or with 2.5 or 3 mM $CuSO_4$. (**b**) OsHMA4 is not involved in transportation of Cd, Co, Ag, Pb, Mn and Zn in yeast. Serial dilutions (1:10) of yeast cultures were spotted on the medium containing without (Control) or with 20 μM $CdCl_2$, 1 mM $CoCl_2$, 2 μM $AgNO_3$, 20 μM $Pb(NO_3)_2$, 10 mM $MnCl_2$ or 1 mM $ZnSO_4$. Pictures were taken after 3 days growth at 30°C.

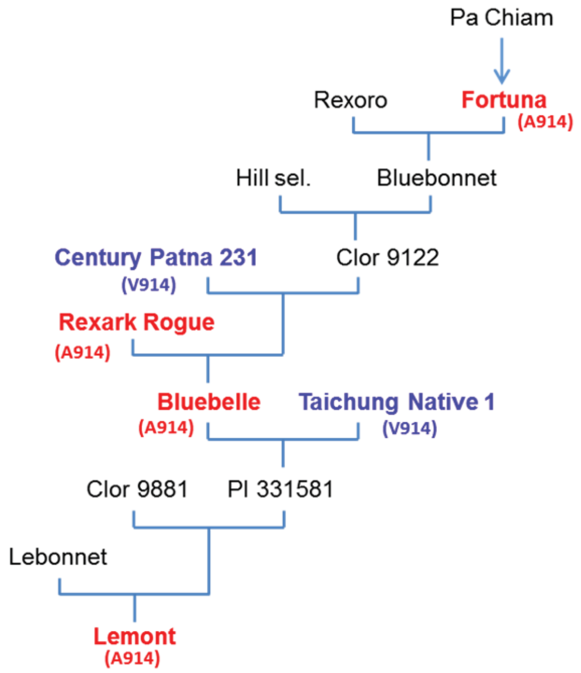

https://npgsweb.ars-grin.gov/gringlobal/search.aspx

Supplementary Figure 14. The pedigree of Lemont. The pedigree was obtained from USDA National Small Grains Collection website https://npgsweb.ars-grin.gov/gringlobal/search.aspx. The functional variant of V914A is shown under the ancestors for which we had data due to their being members of the USDA Core Collection. One likely source of the strong allele of *OsHMA4* in Lemont is its ancestor Pa Chiam, a landrace from Taiwan. The variety Fortuna, which was selected out of this landrace and contains the strong allele of *OsHMA4*, was widely used in breeding in the USA which may explain the enrichment the strong allele among rice accessions from the USA.

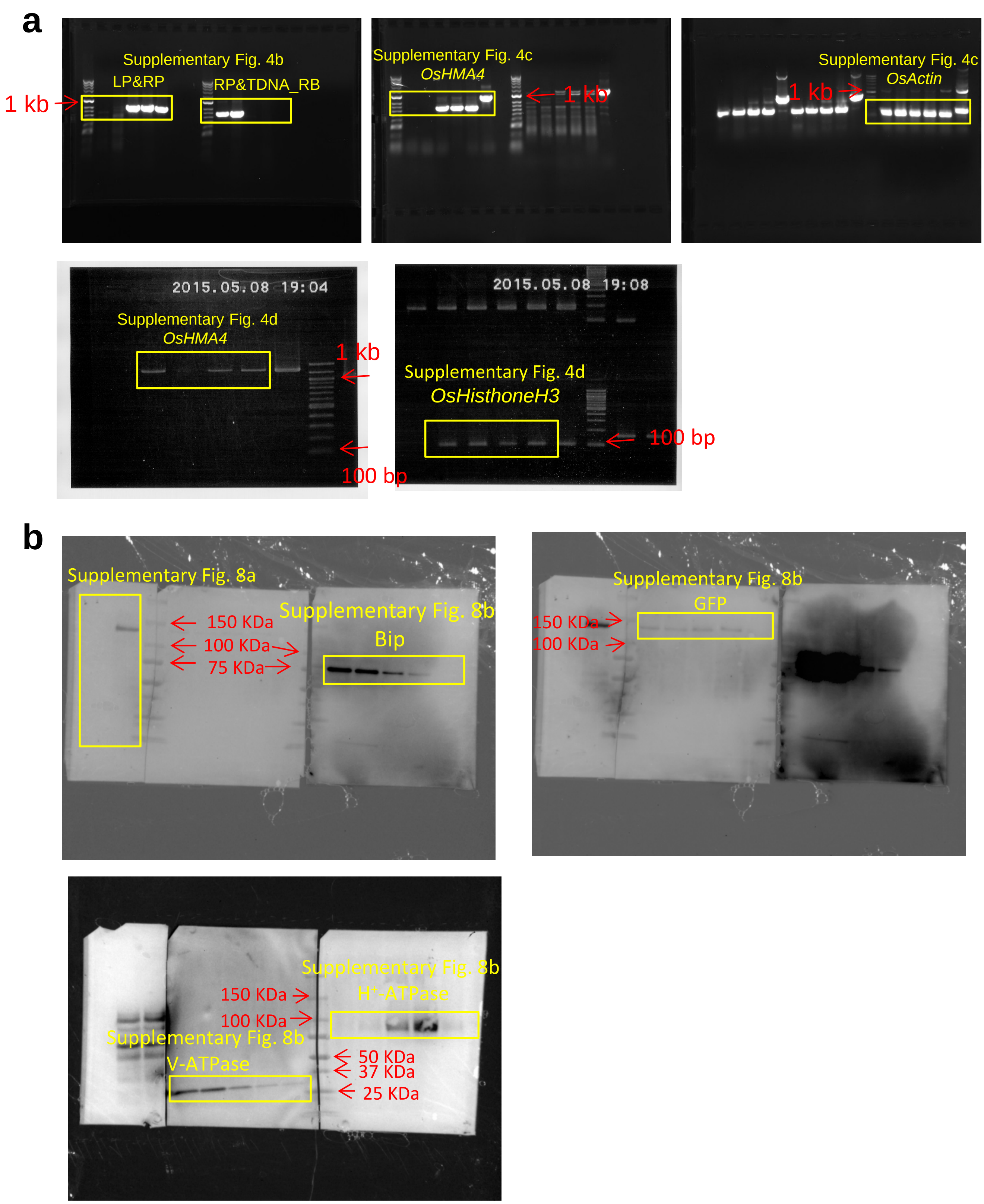

Supplementary Figure 15. Full uncropped versions of gel and western blot images. (**a**) Full uncropped versions of gel images shown in Supplementary Fig. 4b-d. (**b**) Full uncropped versions of western blot images shown in Supplementary Fig. 8a and 8b. The yellow rectangles indicate the cropped area. The red arrows indicate the molecular markers.

Supplementary Table 1. All candidate genes in QTL fine mapping region.

Chromosome	MSU_locus	end5	end3	Annotation
chr02	LOC_Os02g10010	5,207,165	5,208,683	expressed protein
chr02	LOC_Os02g10020	5,209,231	5,212,384	Mad3/BUB1 homology region 1 domain containing protein
chr02	LOC_Os02g10030	5,216,899	5,218,060	hypothetical protein
chr02	LOC_Os02g10040	5,221,797	5,220,249	guanine nucleotide-exchange protein like
chr02	LOC_Os02g10050	5,228,731	5,231,338	expressed protein
chr02	LOC_Os02g10060	5,238,179	5,242,113	SWIRM domain containing protein
chr02	LOC_Os02g10070	5,249,323	5,242,702	citrate synthase
chr02	LOC_Os02g10080	5,250,028	5,253,738	zinc finger C-x8-C-x5-C-x3-H type family protein
chr02	LOC_Os02g10090	5,257,593	5,254,077	expressed protein
chr02	LOC_Os02g10100	5,266,509	5,259,816	leucine-rich repeat receptor protein kinase EXS precursor
chr02	LOC_Os02g10110	5,270,233	5,276,454	Leucine Rich Repeat family protein
chr02	LOC_Os02g10120	5,282,623	5,276,617	lipoxygenase
chr02	LOC_Os02g10130	5,306,435	5,297,643	tubulin binding cofactor C
chr02	LOC_Os02g10140	5,315,119	5,318,064	bZIP transcription factor domain containing protein
chr02	LOC_Os02g10150	5,321,781	5,322,421	bZIP transcription factor
chr02	LOC_Os02g10160	5,328,300	5,324,531	AGAP003371-PA
chr02	LOC_Os02g10170	5,329,016	5,330,521	expressed protein
chr02	LOC_Os02g10180	5,336,986	5,331,894	dnaJ homolog subfamily C member
chr02	LOC_Os02g10190	5,339,007	5,344,517	DUF1680 domain containing protein
chr02	LOC_Os02g10200	5,347,486	5,345,589	zinc finger A20 and AN1 domain-containing stress-associated protein
chr02	LOC_Os02g10210	5,348,720	5,350,221	expressed protein
chr02	LOC_Os02g10220	5,351,467	5,355,147	heat shock protein DnaJ
chr02	LOC_Os02g10230	5,357,310	5,361,634	metal cation transporter
chr02	LOC_Os02g10240	5,366,226	5,364,379	ZOS2-05 - C2H2 zinc finger protein
chr02	LOC_Os02g10250	5,369,277	5,369,552	hypothetical protein
chr02	LOC_Os02g10260	5,380,164	5,373,056	outer membrane protein, OMP85 family protein
chr02	LOC_Os02g10270	5,384,499	5,392,658	retrotransposon protein
chr02	LOC_Os02g10280	5,399,038	5,396,243	transposon protein
chr02	LOC_Os02g10290	5,404,703	5,410,764	copper-transporting ATPase (OsHMA4)
chr02	LOC_Os02g10300	5,411,327	5,414,291	polygalacturonase
chr02	LOC_Os02g10310	5,418,652	5,414,913	fumarylacetoacetase
chr02	LOC_Os02g10320	5,424,926	5,422,121	3-oxoacyl-synthase
chr02	LOC_Os02g10330	5,428,867	5,431,872	retrotransposon protein
chr02	LOC_Os02g10340	5,438,758	5,432,508	retrotransposon protein
chr02	LOC_Os02g10350	5,442,079	5,447,380	MLO domain containing protein
chr02	LOC_Os02g10360	5,453,090	5,451,819	MONOCULM 1
chr02	LOC_Os02g10370	5,464,324	5,464,034	hrpN-interacting protein from Malus
chr02	LOC_Os02g10380	5,468,118	5,465,521	Regulator of chromosome condensation domain containing protein
chr02	LOC_Os02g10390	5,470,496	5,468,367	chlorophyll A-B binding protein
chr02	LOC_Os02g10400	5,475,134	5,470,929	expressed protein
chr02	LOC_Os02g10410	5,475,958	5,475,698	hypothetical protein

Candidate genes were identified from Rice Genome Annotation Project version 6.0 (http://rice.plantbiology.msu.edu/).

Supplementary Table 2. Sequence variation and allele frequency of OsHMA4.

Chr.	Position on genome[a]	Nucleotide		Position in protein	Amino acid		Nipponbare allele frequency		TeQing allele	Lemont allele
		Nipponbare allele	Other allele		Nipponbare allele	Other allele	In 950 worldwide varieties[b]	In USDA core collection[c]		
2	5406703	A	T	104	E	V	0.996233522	1	E	E
2	5406745	A	G	118	K	R	0.996204934	1	K	K
2	5407299	T	C	303	F	L	0.92961165	0.995552261	F	F
2	5407721	T	C	385	F	S	0.996175908	1	F	F
2	5408812	C	T	553	A	V	0.970588235	0.852021358	A	A
2	5409240	T	G	660	S	A	0.973154362	0.914751668	S	S
2	5409481	A	G	704	I	M	0.455319149	0.564954683	I	I
2	5409821	G	A	818	G	S	0.988721805	1	G	G
2	5410205	T	C	914	V	A	0.993174061	0.949203942	**V**	**A**

[a]Position in bp according to IRGSP build 4.0.

[b]Allele frequency from Huang et al., 2011.

[c]Allele frequency calculted by genotyping 1,349 accessions in USDA rice core collection.

Supplementary Table 3. The primers used in this study.

Primer name	Primer sequence (5'-3')	Description
**Primers for fine mapping**		
H24454-L	TGTGACCAGTGCTGCTGAGGA	SSR marker for fine mapping
H24454-R	TCACAGTTGCTCCATTCAGTGCCA	
H24632-L	ACCAGAAACTTGGTGACAGCAAAAGT	SSR marker for fine mapping
H24632-R	GTGGGCAGACTCTTCGTGCCC	
H25078-L	GTTGAGATTCGACGGTCCAT	SSR marker for fine mapping
H25078-R	GGTGGAGAAGAGGGGGTTAG	
H25100-L	CAACGCGAAGCTCTACTGG	SSR marker for fine mapping
H25100-R	GACCCGGCCTTCTACCTAAC	
RM3294-L	ATAGACGGATGGCACGAGATGC	SSR marker for fine mapping
RM3294-R	ATGTGTTCAGTTCAGTGGTGAGTGG	
RM5664-L	GTTCGGCTCCACCTAAACCAAGC	SSR marker for fine mapping
RM5664-R	GGCATTCGTCTCGTCTTTGAGG	
H25397-L	CCGTCAATTCTCGATCGTTT	SSR marker for fine mapping
H25397-R	TCCACATCTCCACAACCTGA	
RM6378-L	CTGATCATCTCATGCCTCCTACG	SSR marker for fine mapping
RM6378-R	TCCATCTCCCAATATGACCAACC	
H25532-L	AAGCTGCGAGCTGTTTGTTT	SSR marker for fine mapping
H25532-R	GAAATCCAGTTGCAGGAAGC	
H25692-L	TCCATCCTTTGCTTCAATCC	SSR marker for fine mapping
H25692-R	GCCACAGCCCATCTTTGTAT	
H25704-L	TCAACGTCTCGCCACAATAA	SSR marker for fine mapping
H25704-R	CATTGTGGATGCCCTTCTTT	
H25735-L	CACAGAAGCCCACAACCTTC	SSR marker for fine mapping
H25735-R	CATGAGCTGCAAAGAGCAAG	
H25854-L	CCTGCATGCTCTCCTCTGGGC	SSR marker for fine mapping
H25854-R	AGACAGTAGCAGCATGGAGAACAGT	
H26652-L	TAACTCAGGCCACCGATCTT	SSR marker for fine mapping
H26652-R	CGTTTGACCGTCCGTCTTAT	

Primers for constructs and expression		
OsHMA4-NOS-F	TCAGGATCCATGGAGCAGAATGGAGAGAACC	Amplify OsHMA4 linked with NOS for transgenic complementation test
OsHMA4-NOS-R	AACAGATCTGATCTAGTAACATAGATGACACCGCG	
pOsHMA4-F	CTGGTACCACTAGTTCAGTACCCAACAGGCAATAAGACTAAG	Amplify OsHMA4 promoter for transgenic complementation test
pOsHMA4-R	CGGGATCCCCCTGTCCCAGGAACAACCAAAAAACC	
pOsHMA4-F	CTGGTACCACTAGTTCAGTACCCAACAGGCAATAAGACTAAG	Amplify coding region of OsHMA4 for transgenic complementation test
GFP-OsHMA4-R2	GGATCCTCACACCAAATCCGGGTCATTCTTG	
OsHMA4-C-F	GATCCGGAATGGAGCAGAATGGAGAGAACC	Amplify OsHMA4 for transgenic complementation test
OsHMA4-C-R	GATCCGGATCACACCAAATCCGGGTCAT	
GFP-OsHMA4-F2	TCTAGACATGGAGCAGAATGGAGAGAACCATCTC	Amplify OsHMA4 for 35S:GFP-OsHMA4 and AtHMA5pro:GFP-OsHMA4 constructs
GFP-OsHMA4-R2	GGATCCTCACACCAAATCCGGGTCATTCTTG	
OsHMA4pro-F	CTGCAGACCCCGTCTCATTTCGCGTGC	Amplify OsHMA4 promoter for promoter GUS consntruct
OsHMA4pro-R	GTCGACTCCCTGTCCCAGGAACAACCAA	
HMA5pro-F	AAAACTGCAGTTCACCGCTGGAACAAT	Amplify AtHMA5 promoter for AtHMA5pro:GFP-OsHMA4 construct
HMA5pro-R	AAAAGTCGACGATCGTCTTGTGCGCGAATC	
YC-OsHMA4-F2	CGCGGATCCAAAAAATGGAGCAGAATGGAGAGAACCATCTC	Amplify OsHMA4 for the yeast construct
YC-OsHMA4-R2	CCGGAATTCTCACACCAAATCCGGGTCATTCTTG	
OsHMA4-RT-F	TGGCAGATAGGATTTCACGGTTT	Primers for RT-PCR on transgenic complementation lines in Supplementary Fig. 4d
OsHMA4-RT-R	TCACACCAAATCCGGGTCAT	
OsHistoneH3-F	AGTTTGGTCGCTCTCGATTTCG	Primers for RT-PCR on transgenic complementation lines in Supplementary Fig. 4d
OsHistoneH3-R	TCAACAAGTTGACCACGTCACG	
OsHMA4qRT-F2	CTGCTGGTGCAGAGGCGAACAG	Primers for qRT-PCR in Fig. 3e-g and Supplementary Fig. 7b,c
OsHMA4qRT-R2	CACTCACCCCTGCCCCTGGAT	
Act-rts	TGGTCGTACCACAGGTATTGTGTT	Primers for qRT-PCR in Fig. 3e-g and Supplementary Fig. 7b,c
Act-rta	AAGGTCGAGACGAAGGATAGCAT	
OsHMA4qRT-F1	TGGCTGCTGGACCCAAGAATG	Primers for qRT-PCR in Fig. 3a, b
OsHMA4qRT-R1	AGTCAGGAGGGCCACTGCAAAC	
OsHistoneH3qRT-F	AGTTTGGTCGCTCTCGATTTCG	Primers for qRT-PCR in Fig. 3a, b
OsHistoneH3qRT-R	TCAACAAGTTGACCACGTCACG	
COPT2qRT-F2	CGCCGGCTATGGCGTTGGTT	Primers for qRT-PCR in Supplementary Fig. 9d
COPT2qRT-R2	GCAGCCTGAAGACGGCGGAA	
FSD1qRT-F2	GGATTCACAGCATTGGGAGT	Primers for qRT-PCR in Supplementary Fig. 9d
FSD1qRT-R2	TGGAGGAAAACCATCAGGAG	
ZIP2qRT-F1	CGCTTGGAGAAACCTATGGA	Primers for qRT-PCR in Supplementary Fig. 9d
ZIP2qRT-R1	CGACACCTATGGGACTCGAT	
SPL7qRT-F1	CAGGCAGACTGTTCACCAGA	Primers for qRT-PCR in

**Primers from genotyping USDA rice core collection**		
OsHMA4snp104-F3	CCTCAATTTCGAGGTCGAAG	dCAPS marker (*Sca* I) for genotyping the polymorphic site of E104V
OsHMA4snp104-R3	AGCTTGTACATGCCATTCCTTT	
OsHMA4snp118-F2	AAGAAGCTATCGAAGGCCTCAATTT	dCAPS marker (*Hinf* I) for genotyping the polymorphic site of K118R
OsHMA4snp118-R2	AGCAGCTTGTACATGCCATGACT	
OsHMA4snp303-F	AGCGCCATCATGAAATTAGG	dCAPS marker (*Xho* I) for genotyping the polymorphic site of F303L
OsHMA4snp303-R	GTAAAACAGCCAATCCTCGA	
OsHMA4snp385-F	CAGAGTCATTTGAAGGACAGAATT	dCAPS marker (*EcoR* I) for genotyping the polymorphic site of F385S
OsHMA4snp385-R	CTCCAGATATTTCCCCAGCA	
OsHMA4snp553-F2	CATGCATACTTTCTGTGAGATCG	dCAPS marker (*Pst* I) for genotyping the polymorphic site of A553V
OsHMA4snp553-R2	CCAAGCCATGTAAGAACTGCA	
OsHMA4snp660-F2	CTTCTGTTGTTCAAACAAAGGTGTGC	dCAPS marker (*Hha* I) for genotyping the polymorphic site of S660A
OsHMA4snp660-R2	TGCATCTAAGAAGCCAACCTCT	
OsHMA4snp704-F4	ACTTTGTCAGGCGAACAGTGAGCATCC TC	dCAPS marker (*EcoR* V) for genotyping the polymorphic site of I704M
OsHMA4snp704-R4	TGGATGCACCTCAAAGTCCTTGGACGA TAT	
OsHMA4snp818-F	CGTGCCATTTCATACCTCAG	dCAPS marker (*Pvu* II) for genotyping the polymorphic site of G818S
OsHMA4snp818-R	GATCTCGGCAAATACTCAGC	
OsHMA4snp914-F2	GGGCTCTGGGCTACGACT	dCAPS marker (*Hinf* I) for genotyping the polymorphic site of V914A
OsHMA4snp914-R	GAGCAGACGACGCTCACC	
